# High‐Density *E. Coli* Cultivation Arrays for Combinatorial Drug Testing With Subsequent Lipid Profiling by MALDI‐MS

**DOI:** 10.1002/advs.76678

**Published:** 2026-07-23

**Authors:** M. Breitfeld, C. L. Dietsche, F‐L. Born, I. Onipko, L. Blaase, P. S. Dittrich

**Affiliations:** ^1^ Department of Biosystems Science and Engineering ETH Zürich Basel Switzerland

**Keywords:** antibiotic susceptibility testing, combinatorial drug testing, droplet microfluidics, lipid profiling, MALDI‐MS, microarrays

## Abstract

The increasing resistance of bacteria to current drugs underscores the urgent need for new antibiotics. Combination therapies offer an alternative strategy by enhancing efficacy and delaying resistance, with synergies assessed in checkerboard assays using systematically varied drug concentrations. Here, we introduce a miniaturized checkerboard assay for high‐throughput combinatorial antibiotic testing against *E. coli*. Our platform reduces sample volume significantly, achieves high sample densities of 20832 droplets per microscopy slide, and allows generation of checkerboard arrays in under 25 min prior to incubation and analysis. We established the platform by comparing *E. coli* growth across different culture volumes and performed assays to determine the minimum inhibitory concentration (MIC) for various drugs. Further, we replicated the synergistic effect of amoxicillin/clavulanate against an amoxicillin‐resistant strain. Testing various antibiotic combinations allowed us to determine fractional inhibitory concentration indices (FICI) of up to 1.86 ± 0.42, suggesting indifferent effects with a tendency towards antagonism. Lipidomic profiling of *E. coli* revealed the characteristic lipid composition of primarily phosphatidylethanolamines (PE) and phosphatidylglycerol (PG). We identified PE32:0 that showed enhanced signal intensities at the MIC region, hinting at stress‐induced lipid changes. Our platform holds promise for exploring combinatorial drug effects across cell types, with unique capabilities for simultaneous cell monitoring and MALDI‐MS analysis.

## Graphical abstract

High‐throughput antibiotic cross‐gradient assays on microdroplet arrays. A multichannel printhead together with five pressure regulators forms droplet arrays of up to 20 832 droplets within 25 minutes. Each droplet contains unique concentrations of antibiotics, allowing to moniture a vast spectrum of bacterial repsonses over night.  The bacterial growth inside the droplets is imaged by time‐lapse microscopy and their lipids further profiled by MALDI‐MS.

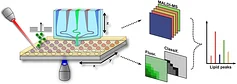

## 1 Introduction

Antibiotic resistance is a global challenge. It is estimated that by 2050, 1.9 million deaths will be directly attributed to antibiotic resistance, and 8.2 million deaths will be associated with it [1]. In contrast, the rate at which the FDA approves new drugs has steadily slowed down over the past 50 years, with most approved drugs being analogs of older ones [2, 3]. Even when new antibacterial drugs are developed, bacteria rapidly evolve to develop resistance. An alternative approach to discovering new antibiotics is combination therapy, where patients are treated with two or more drugs to combat the infection. These drug combinations can improve efficacy, limit antibiotic resistance, decrease required dosages, and broaden antibacterial coverage [4]. Drug combinations have been effectively used to treat diseases like cancer [5], tuberculosis [6], and malaria [7]. However, for antibiotic resistance, further exploration is needed, though exceptions exist, such as the discovery of beta‐lactamase inhibitors (e.g., clavulanate), which is commonly paired with amoxicillin to combat resistant bacteria [8].

Identifying drug interactions often involves phenotypic screenings across extensive combinatorial chemical libraries. This process also includes identifying resistance mechanisms through sequencing or advanced analytical techniques like mass spectrometry. For instance, recent lipidomic analyses using mass spectrometry gave insights into lipid‐based signatures that may explain certain resistance mechanisms [9, 10, 11, 12]. While mass spectrometry enables biochemical profiling, functional validation of interactions, such as the effects of combining drugs, often relies on traditional assays. Checkerboard assays, commonly used to test drug interactions, are typically conducted in 96‐ or 384‐well plates but are resource‐intensive, particularly when exploring wide concentration ranges or multiple drug combinations. Automated liquid handling can enhance throughput and reliability, yet its efficiency decreases with complex checkerboard setups and requires substantial reagent volumes, while reagent volumes in the microliter range or smaller are prone to rapid evaporation [13, 14].

Miniaturized versions of well plates, called microdroplet arrays, could be a promising alternative due to the enormous reduction of sample volume and increase in throughput [15]. These platforms offer conveniently accessible pico‐to nanoliter water‐in‐oil droplets at distinct positions, similar to well plates. The accessibility from the top allows the droplets to be deterministically sampled using custom 3‐axis liquid‐handling robots, similar to pipetting robots. Additionally, detection methods beyond fluorescence microscopy, namely mass spectrometry, can be employed to further evaluate the droplet content [16, 17, 18]. Microdroplet arrays have been successfully applied for screening of (bio‐)chemical libraries [19], forming chemical gradients [20, 21, 22, 23], and conducting cell assays [18, 24, 25].

So far, their potential to study combinatorial antibiotic drug effects and resistance mechanisms has not yet been exploited, as the throughput decreases significantly with complex combinatorial assays.

In this study, we overcome limitations in the throughput and present a novel approach to perform microdroplet array‐based checkerboard assays for antibiotic drug testing with subsequent lipid profiling of *E. coli*. We are building up on our previously published approach to quickly generate microdroplet arrays [26]. In brief, a pressure pump continuously transfers liquid from Eppendorf tubes through a thin capillary onto a patterned glass plate with hydrophilic areas (referred to as “spots”) that is placed in an oil bath. Moving the glass plate in close proximity to the capillary outlet shears off droplets that adhere to the spots. Here, we have further expanded the system by integrating a multichannel printhead connected to five individual pressure regulators that allows the formation of complex combinations immediately before a droplet is formed [27]. In other words, in this advanced system, we create all combinations of drugs and concentrations in one run, instead of subsequent deposition procedures.

This new technology opens the way to perform a high‐throughput cross‐gradient assay to determine the change in antibacterial efficacy of a combination of antibiotics relative to their individual activity. This comparison is evaluated by observing the growth pattern of *E. coli* using customized image analysis and determining the fractional inhibitory concentration index (FICI). The index is used to categorize the interactions between two drugs and is determined by dividing each drug's minimum inhibitory concentration (MIC) in combination by each drug's MIC alone. Finally, we assess the lipid profile of *E. coli* by MALDI‐MS directly on the microdroplet array without the need for sample preparation or purification steps.

## 2 Results and Discussion

### 2.1 Cross‐Gradient Assay Workflow for Microdroplet Arrays

Our platform to create microdroplet arrays is built on a glass plate. The plate is sputtered with a 300 nm thin layer of indium tin oxide (ITO). The ITO is transparent as required for employing fluorescence microscopy and conductive, as required for MALDI‐MS measurements. Afterwards, the plate is covered with a hydrophobic layer and round hydrophilic spots are created by photolithography. The hydrophilic spots are the anchor sites for the aqueous droplets. Droplets are physically separated by the fluorinated oil phase and hydrophobic boundaries, preventing direct contact between neighboring droplets. Since no surfactants were used, diffusion‐driven cross‐contamination is expected to be minimal. Long‐term stability of the droplet arrays [27] as well as the diffusion time within droplets [28] have previously been demonstrated. For all experiments, we use a plate with 20 832 hydrophilic spots with a diameter of 125 µm and a spot‐to‐spot distance of 225 µm.

The workflow of the high‐throughput cross‐gradient assay is depicted in Figure 1A. First, two antibiotic stock solutions, media, and cells are prepared in Eppendorf tubes. Five individual controllable pressure pumps transfer the reagents into a polydimethylsiloxane (PDMS) microfluidic device (the “printhead”). Inside the printhead, the reagents are mixed based on the applied pressure ratios and transferred to the outlet. Simultaneously, the hydrophobic/hydrophilic patterned microarray moves in close proximity to the outlet and continuously shears off droplets from the aqueous stream that immediately adhere to the hydrophilic spots (Figure 1A‐i). During the course of the microdroplet array formation, each inlet pressure is changed according to the following pattern: constant pressure for the cell solution, increase in antibiotic A over the rows, increase in antibiotic B over the columns, and decrease of the buffer, respectively. Initial droplet evaporation is prevented due to a perfluorinated oil bath. For the subsequent time‐lapse imaging steps, small water trays are placed inside the oil bath, and the tray is sealed with adhesive breathable PCR plate foil. This arrangement effectively prevents evaporation (Figure S1) of both the oil and the microdroplets and still allows gas exchange. During the long‐term imaging and incubation on an epifluorescence microscope, the growth of bacteria is monitored across the microdroplet arrays (Figure 1A‐ii). After one night of imaging, the oil is decanted to allow drying of the droplets. Afterwards, a layer of matrix 2,5‐dihydroxybenzoic acid (DHB) is sublimated onto the microarray, and mass spectra in the range from 600 to 3000 m/z are recorded for every spot in a MALDI‐TOF/TOF mass spectrometer (Figure 1A‐iii). Figure 1B illustrates the multimodal workflow used to identify the most prominent mass peaks from the untargeted MALDI‐MS analysis. The fluorescence intensity of green‐fluorescent bacteria is utilized to determine their respective MIC values and to classify the bacterial state into live, dead, and MIC. Random Forest classification was then applied as a learning algorithm to the MALDI‐MS data to reveal the mass peaks with the highest importance score. The respective peaks are further cross‐referenced with the LIPID MAPS database to identify glycerophospholipids.

**FIGURE 1:**
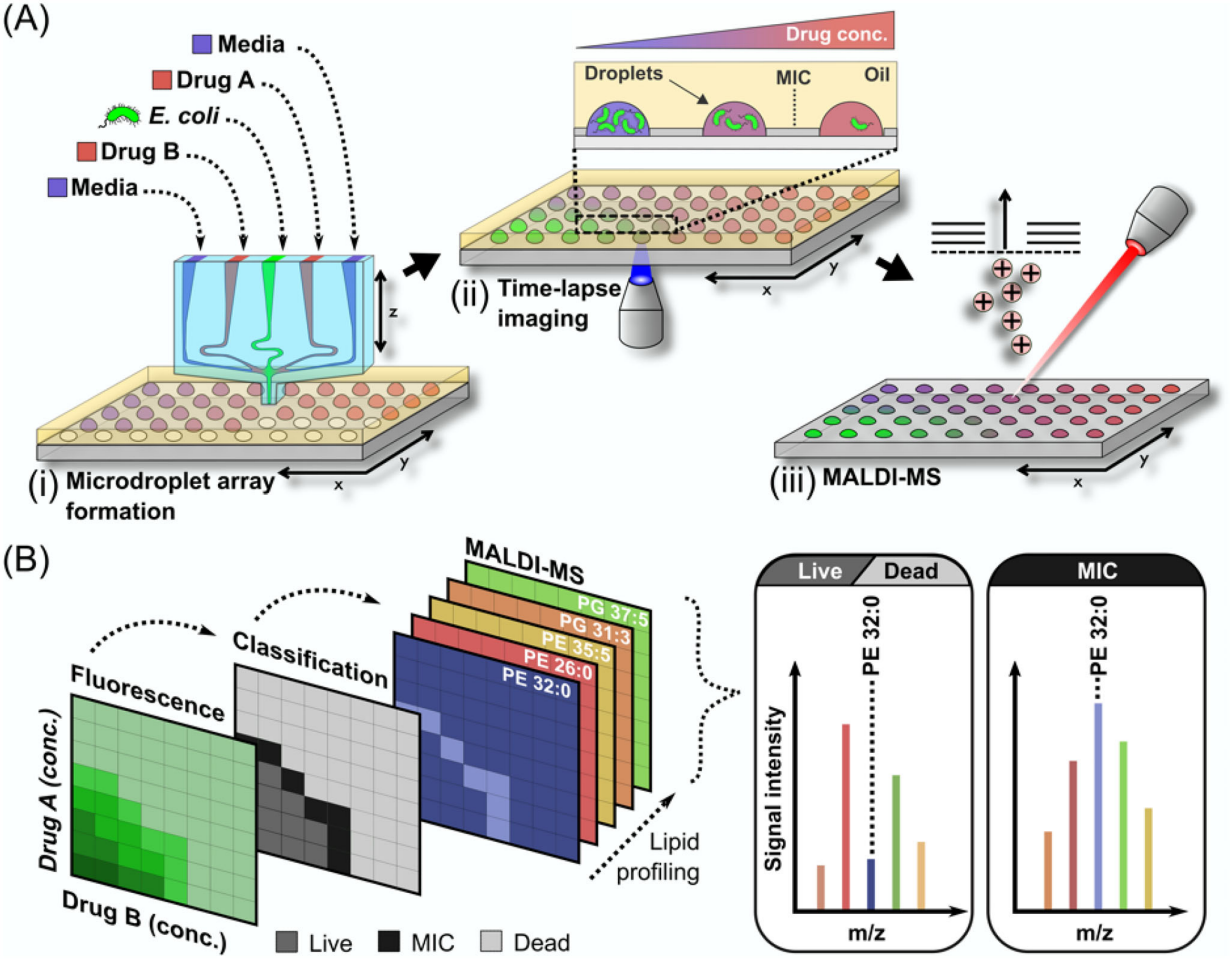
Overview of the high‐throughput cross‐gradient assay performed on microdroplet arrays. (A,i) Formation of the microdroplet array. A multichannel printhead is used to mix and supply the sample. Moving the glass plate near the printhead outlet results in shearing off the sample stream and in the formation of the droplets on the microarray. The plate is placed in an oil bath to prevent evaporation and mounted on a motorized microscopy stage. Here, we use a printhead with five inlets together with five pressure regulators to create droplets that contain unique concentrations of two antibiotics while maintaining a constant cell concentration. (ii) The bacterial growth inside the droplets is imaged by time‐lapse microscopy and further analyzed by (iii) MALDI‐MS. (B) Based on the fluorescence signal, bacteria are classified as dead or alive. Inhibited growth can be observed in droplets filled with antibiotics near the minimum inhibitory concentration (MIC). This designation is used to narrow down the untargeted MALDI‐MS analysis and uncover outstanding lipids from the vast bacterial lipid landscape.

Before we applied the entire procedure, we confirmed the performance of the printhead and characterized the bacterial growth in droplets under constant conditions across the plate, with and without drug addition, which is described in the following.

### 2.2 Droplet Printhead Characterization and Cross‐Gradient Plate‐Layout

The applied pressure ratios determine the composition and the volume of the droplet. For simplicity and robust control, all capillaries from tube to printhead and all supply channels in the printhead should have the same hydrodynamic resistance. We reviewed the equal pressure‐volume relation by generating droplets by pressurizing only one inlet at a time. This step was then repeated for all inlet channels (Figure 2A). The solution contained a fluorescent dye, and we considered the fluorescence intensity (integrated per spot) as a means for droplet volume.

**FIGURE 2:**
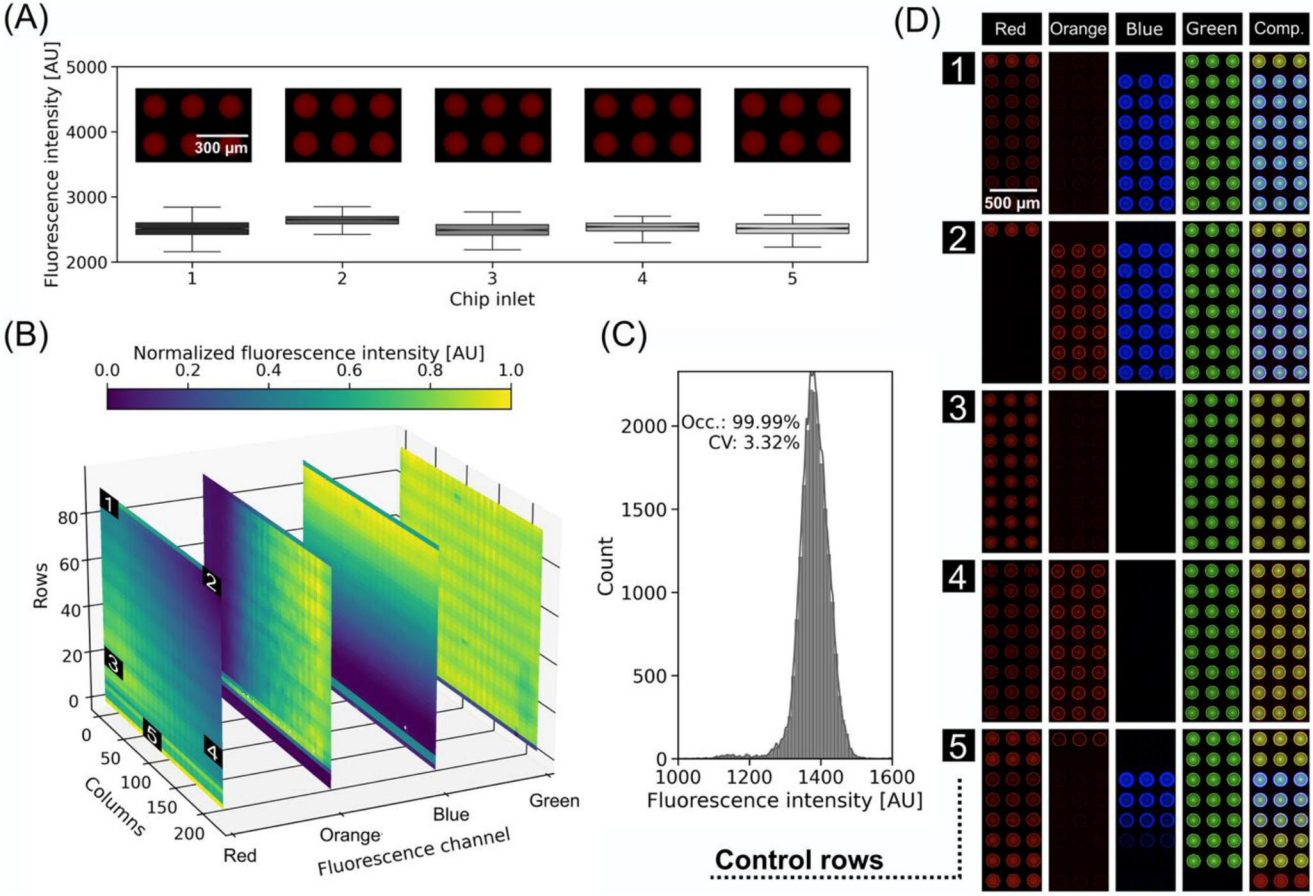
Multichannel printhead characterization and formation of cross‐gradients, here illustrated with fluorescent dyes. (A) Fluorescence images and intensities of droplets generated by activating one inlet channel at a time (n = 672 per condition), yielding mean intensities of 2479.74–2639.58 with standard deviations of 99.70–193.56 and coefficients of variation of 3.78–7.73%, indicating robust and reproducible droplet formation. (B) Heatmaps showing the normalized fluorescence intensities of the 20 832 droplets on the plate containing four different fluorescent dyes (red: dextran Alexa Fluor 647; orange: sulforhodamine B; blue: dextran Cascade Blue and green: fluorescein). The combination of the dyes represents the layout for the cross‐gradient assays. (C) Distribution of the fluorescence intensities of droplets for the constant pressure condition of the green fluorescence (fluorescein) depicted in B (n = 20 608). (D) Fluorescence images showing five different regions of the droplet microarray depicted in B. Region 1–4 highlights the corners of the cross‐gradient and region 5 the concentration steps for the controls. The control image presented here contains five concentration steps of dextran Cascade blue (“blue”) and the complementary dextran Alexa Fluor 647 (“red”). The first control row of droplets does not contain any fluorescein and is therefore excluded from the occupancy estimation in (C).

Comparison of the average fluorescence intensities of all droplets for each inlet channel confirmed equal performance of the involved capillaries and microfluidic structures (Figure 2A). Next, we deposited droplets on the plate containinga mixture offour fluorescent dyes byutlizing all five microfluidic channels. Figure 2B shows the assay layout that is used throughout all cross‐gradient experiments (side‐by‐side in Figure S2A). For visualization and optimization, we first used four different dyes (inlet channels 1,5: red‐fluorescent dextran Alexa Fluor 647; inlet channel 2: orange‐fluorescent sulforhodamine B; inlet channel 4: blue‐fluorescent dextran Cascade Blue and inlet channel 3: green‐fluorescent fluorescein), which corresponds in the combinatorial assays (Section 2.5) to the supply of buffer (inlet channels 1,5), antibiotic compounds A and B (inlet channels 2,4) and bacteria (inlet channel 3). The false‐color images show the normalized fluorescence intensities of the 20 832 droplets on the plate. The combination of the dyes led to a unique concentration combination for every droplet (enlarged image in Figure S3). This was further visualized in the fluorescence images from different regions of the microdroplet array (Figure 2D), here optically resolved for the four different fluorescence colors of the dyes. We could create fine‐tuned gradients across the plate, as well as sharp concentration steps, e.g. to implement controls, as can be seen in region 5 (Figure 2D). The corresponding fluorescence‐concentration gradients for Figure 2, including both the cross‐gradient and the control rows, are shown in Figure S2B,C, demonstrating linear behavior up to the limit of detection (LOD), which depends on the dye and imaging magnification. The lower pressure regime, which is partially masked by the LOD in the present measurements, was previously investigated in detail [27], and was likewise found to exhibit a linear correlation between concentration and fluorescence intensity. In that study, the accuracy of pressure‐controlled mixing and droplet composition was validated, showing a strong linear correlation (R^2^ = 0.998) between concentration and measured fluorescence intensities. We confirmed homogeneous droplet generation with a low coefficient of variation (3.32%) and achieved a high droplet occupancy of 99.99% (Figure 2C and Figure S4), respectively, for the green fluorescence intensities of fluorescein (Figure 2B). Most importantly, we were able to generate the highly dense microdroplet array within 25 min, illustrating that the combinatorial complexity could be achieved without compromising throughput. The short time is crucial for the following application, where the imaging should start as fast as possible for monitoring bacterial growth curves, given the rapid doubling times of *E. coli*.

### 2.3 Bacterial Growth on Microdroplet Arrays

Next, we characterized the bacterial growth of the *E. coli* strain ATCC 25922 on our microdroplet array. The strain produces intracellular superfolder green fluorescent protein (sfGFP) that we use as a marker to monitor the bacterial growth on a fluorescence microscope. We generated an array of 20 832 droplets containing *E. coli* at an optical density (OD_600_) of 0.01, resulting in an estimated average cell number of 5 cells/droplets with a droplet volume of ∼500 pL. We then imaged over the course of 12 h the bacterial growth (i.e. the production in sfGFP) inside the droplets (Figure 3A). We evaluated the images by a custom MATLAB code that detected the fluorescence signal of the bacteria (Figure S5). Analysis of the first time point directly after droplet generation (t = 0) confirmed the theoretically estimated cell distribution inside the droplets (Figure 3B). Importantly, by visualizing the cell distribution across the entire plate (Figure 3C), no cell gradient or other pattern across the plate was observed. The homogeneous distribution underlined that, within the 25 min of the microdroplet array formation, the bacteria were neither starting to sediment inside the Eppendorf tube, nor blocking the channels of the printhead. Figure 3D visualizes the respective growth curves of the experiment. In total, we could record growth in 18 210 out of 20 832 droplets (87.4% occupancy obtained from the last time point, Figure S6). By grouping and averaging the growth curves based on the initial number of cells per droplet, which arises from Poisson‐distributed loading during droplet formation, we found that droplets containing fewer cells exhibit a longer lag‐phase and lower fluorescence intensity at the stationary phase (Figure 3E). The delay in lag ‐phase, common in microfluidic volumes, can also be perceived as an extreme manifestation of the inoculum effect. According to this pattern, a decrease in the initial number of cells increases the time of adaptation of bacteria to the new environment [29]. However, the difference in fluorescence intensity at the stationary phase was rather surprising, since we expect similar cell densities in the droplet at the end of the exponential growth phase. We speculate that in smaller populations, cells might prioritize basic survival and growth over protein expression during the lag and exponential phases. This could lead to less overall sfGFP accumulation by the time they reach stationary phase.

**FIGURE 3:**
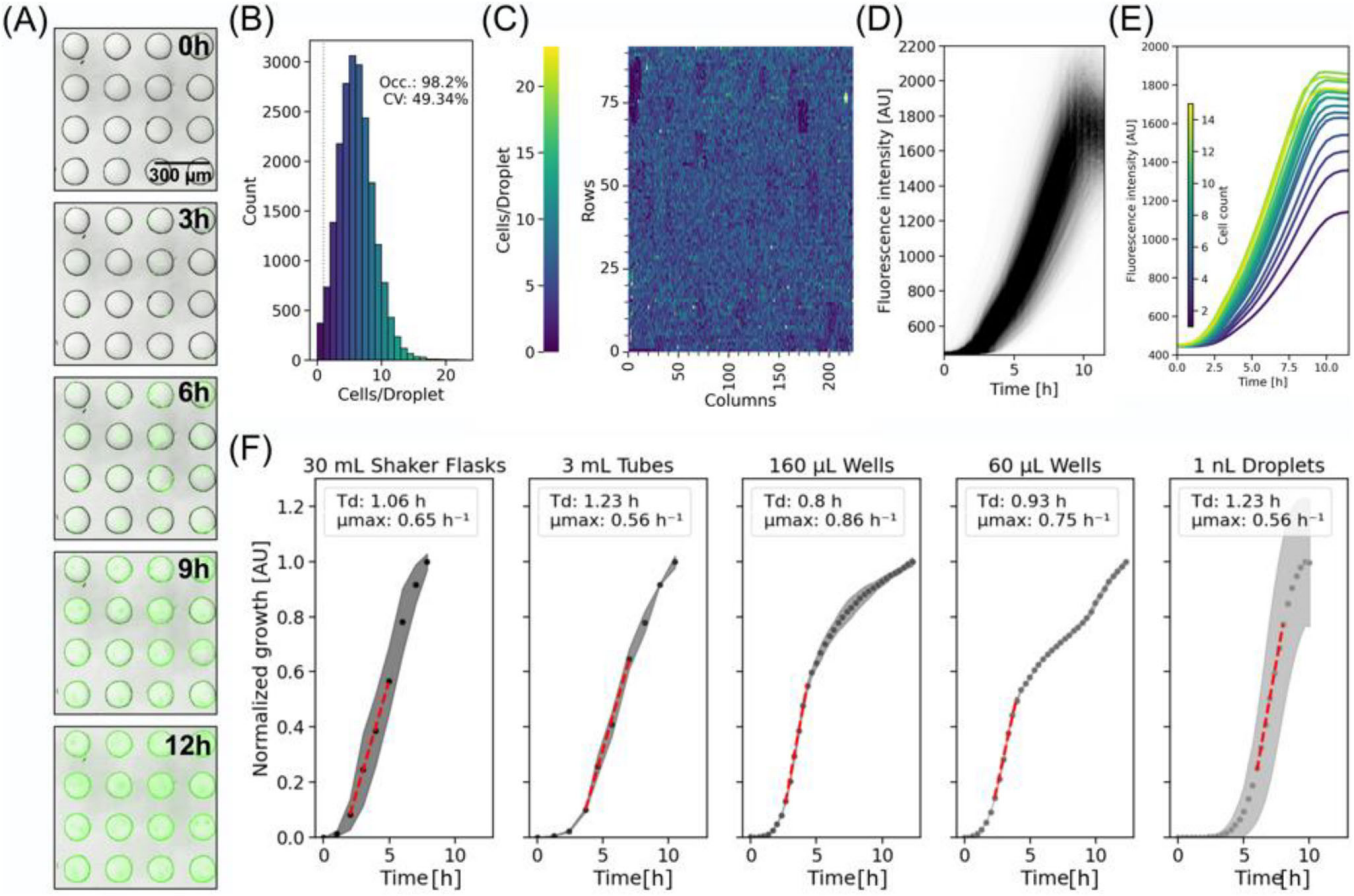
Growth characterization of *E. coli* on the microdroplet array. (A) Time‐lapse overlaid brightfield and fluorescence images of droplets containing green‐fluorescent *E. coli*. (B) The histogram shows the distribution of the starting cell number per droplet (n = 18 210 droplets) and (C) the corresponding heatmap. (D) Visualization of 18 210 *E. coli* growth curves obtained on one microdroplet array (one growth curve per droplet). (E) Growth curves from D were grouped and averaged according to initial cell number (cell spreading follows the Poisson distribution). (F) Comparison of *E. coli* growth curves in five different culture volumes (30 mL shaker flasks, 3 mL tubes, 160 µL 96 well plate, 60 µL 384 well plate and 1 nL microdroplet array). Doubling times (Td) and growth rates (µmax) of the exponential phases are depicted in the legends.

Finally, we performed growth experiments of *E. coli* in different culture volumes. For this test series, we selected a broad variety of culture vessels, starting from 30 mL cultures in 250 mL shaker flasks, 3 mL cultures in 10 mL round‐bottom test tubes, 160 µL cultures in 96 well‐plates and 60 µL cultures in 384 well‐plates, as well as 1 nL cultures on our droplet microarray. By comparing the growth curves of *E. coli* throughout the different culture vessels (Figure 3F), it is noticeable that the bacteria inside the shaker flask show a 1‐h shorter lag phase and therefore enter the exponential phase faster compared to all other conditions. This was expected due to the higher oxygen transfer into the media promoted by the mixing baffles embedded in the flasks. In contrast, the growth curves inside the droplets exhibited a longer lag phase compared to all other conditions, influenced by two factors: first, the previously mentioned inoculum effect, and second, the measurement method. Bacterial growth in all other conditions was assessed using absorbance, which accounted for a >1 h shorter lag time (Figure S7) compared to fluorescence‐based readouts. This difference can be attributed to the maturation time of the sfGFP after cell division and the dilution of fluorescent protein during cell division, which can delay fluorescence‐based growth detection [30]. Doubling times (0.8–1.23 h) and growth rates (0.86–0.56 h^−^^1^) during the exponential phase showed overall good agreement across culture volumes. While statistical differences were observed between droplets and well plate formats, no significant differences were detected between larger‐scale cultures (Figure S8). However, the doubling times observed in our experiments are generally longer than the optimal *E. coli* doubling times reported in the literature of ∼20 min [31] across all culture conditions. This can be attributed to the fact that bacteria were cultivated under conditions optimized for antimicrobial susceptibility testing rather than maximal growth rate. Furthermore, the use of reporter strains and antibiotic selection may contribute to slightly increased doubling times compared to literature values obtained under optimized growth conditions.

In summary, the bacterial growth in our platform is comparable to the standard methods with an additional advantage of obtaining an unprecedentedly large number of growth curves in parallel with little consumption of medium and cells. For example, using approximately 25 µL cell suspension and within only one night of imaging on a microscope, we can record 18 210 individual growth curves (Figure 3D). This corresponds to >47 plates with 384 wells, which can be imaged only one after another in standard plate readers.

### 2.4 Monitoring Bacterial Growth Under Antibiotic Treatment

Next, we characterized the growth of *E. coli* for a small library of 8 common antibiotics and one antimicrobial peptide (polymyxin B). For each drug, we tested 5 different concentrations (0.25, 0.5, 1, 2, 4x MIC, Figure S9) according to the MIC values given by the European Committee on Antibiotic Susceptibility Testing (EUCAST [32], Figure S9), leading to a total of 46 conditions (including control growth without antibiotics). For each condition, we generated 90 droplets, all deposited on the same microdroplet array, and imaged the bacterial growth over the course of 11 h. Again, we used the fluorescence intensity per droplet as a means for cell growth. For each antibiotic and concentration, we obtained growth curves through the overnight time‐lapse imaging (Figure 4A and Figure S10). We observed that for the majority of antibiotics, growth deviation >5% from the control occurred immediately after the lag phase, approximately 1.5 h after incubation (Figure S11). Further, we deduced the final growth state in percent (Figure 4B) by comparing the fluorescence intensity of the drug‐exposed bacteria to the control after 11 h of growth. The results demonstrate that the MIC assay performed on our platform generally aligns with the recommended MIC values from EUCAST. However, some antibiotics, such as meropenem, ampicillin, and the antimicrobial peptide polymyxin B, may require adjustments of the tested concentration ranges if further studies are conducted. For meropenem and ampicillin, complete growth inhibition was not observed within the tested concentration ranges, likely reflecting that MIC values are determined using highly standardized protocols and reference systems under controlled conditions. For polymyxin B, for which no official EUCAST breakpoint exists, broader initial screening ranges would be required, as the chosen concentrations were comparatively high. Finally, we performed a 96‐well plate MIC assay using a plate reader (Figure S12) and obtained comparable results, suggesting that the observed deviations are not specific to the droplet format but are more likely attributable to factors such as stock solution preparation, dilution accuracy, and pipetting variability. It should also be noted that MIC values are inherently subject to biological and technical variability and are typically reported in discrete dilution ranges rather than as fixed values. Nevertheless, to be consistent with the EUCAST guidelines, we did not modify the MICs for our system in the combinatorial drug testing described in the next chapter. Instead, we selected antibiotics that effectively inhibit bacterial growth within the in Figure 4B established MIC ranges (≤ 1% growth compared to the control).

**FIGURE 4:**
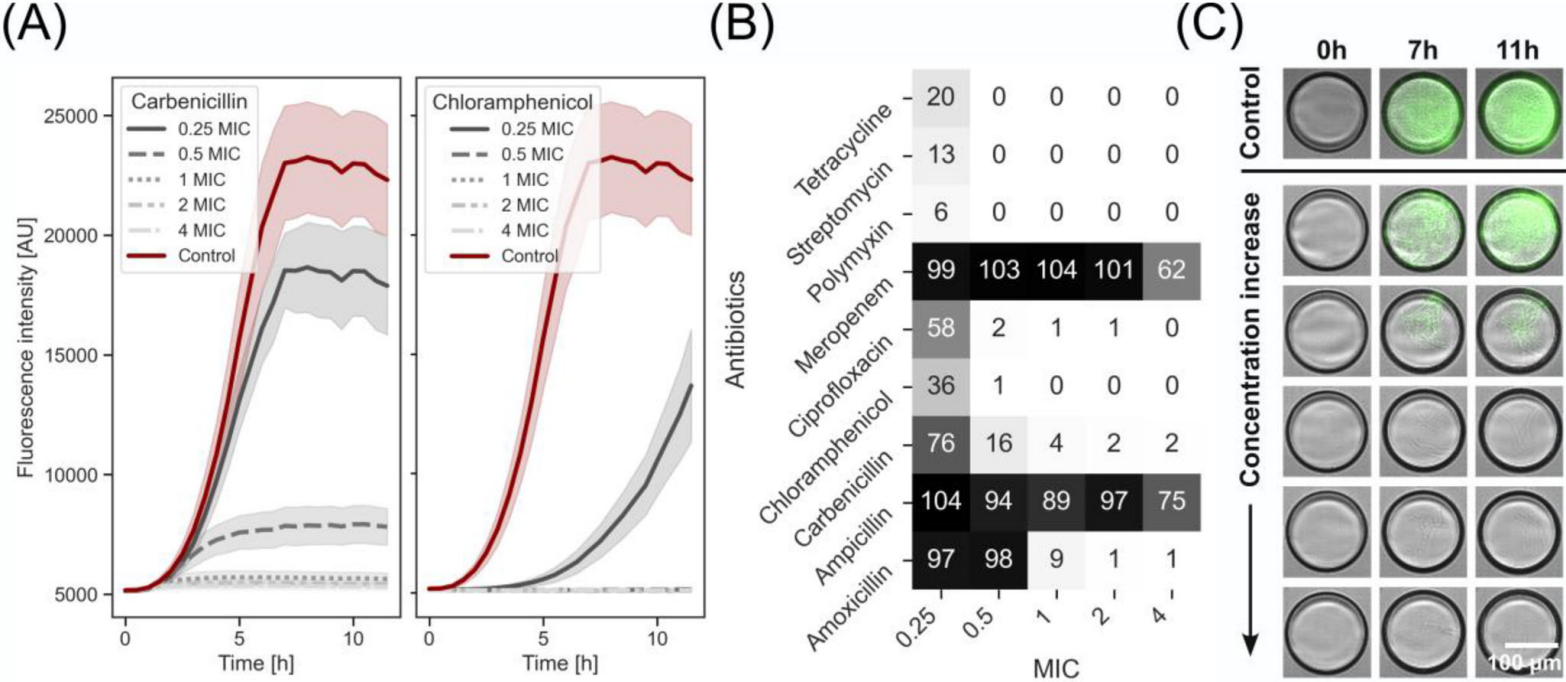
Antibiotic treatment of *E. coli* on microdroplet arrays. (A) Bacterial growth curves in droplets at five antibiotic concentrations (0.25 MIC to 4 MIC) of carbenicillin (bactericidal) and chloramphenicol (bacteriostatic) (n = 90 per condition). (B) The final growth state (%) for every condition was obtained in reference to the control (red line in A). All growth curves are shown in Figure S10. Each entry in the heatmap is averaged from 90 droplets. (C) Corresponding time‐lapse brightfield‐fluorescence images of droplets containing bacteria grown in media and under the exposure of five concentrations (0.25 MIC to 4 MIC) of carbenicillin.

Due to the considerably smaller height of the droplets on the microarray (∼65 µm), compared to the well depth in multi‐well plates (10,72 mm for 384‐well plates) it is possible to gain additional information on morphological changes, as an indicator for cellular response to antibiotic treatment. Example brightfield‐fluorescence images in Figure 4C show elongated *E. coli* at carbenicillin concentrations around the MIC (0.25 MIC to 4 MIC). Carbenicillin is known to cause elongation (filamentous growth) of the cells and the induction of SOS responses [33]. Filamentous growth in *E. coli* under certain antibiotics occurs because the drugs interfere with divisome proteins [34], inhibiting septal peptidoglycan synthesis while allowing continued elongation [35].

### 2.5 Combinatorial Drug Testing and FICI Determination

Next, we determined the combinatorial effects of two compounds. We chose first the pair amoxicillin/clavulanate, which is a commonly used broad‐spectrum beta‐lactam/beta‐lactamase inhibitor combination with activity against Gram‐negative and Gram‐positive bacteria. Clavulanate enhances amoxicillin's effectiveness by inhibiting beta‐lactamases, which would otherwise degrade amoxicillin, allowing it to remain active. EUCAST recommends using a fixed concentration of 2 mg/L clavulanate for susceptibility testing of the amoxicillin/clavulanate combination. Here, we used the amoxicillin‐resistant *E. coli* strain ATCC 35218 and applied the concentration pattern as introduced in Figure 2B, with an amoxicillin gradient over the rows from 0 to 16 mg/L and a clavulanate gradient over the columns from 0 to 8 mg/L. Simultaneously, antibiotic‐free Mueller‐Hinton Broth II (MHB II) media was added to form an opposing gradient over the rows and columns to ensure the same droplet volume across the plate. Additionally, bacteria inside the Eppendorf tube with a starting OD_600_ of 0.25 were diluted in the droplet printhead to reach a final concentration inside the droplets of OD_600_ 0.05 (20 cells/droplet). The typical synergistic growth pattern (fluorescence intensity) of the bacteria across the plate is represented in the 4 heatmaps in Figure 5A (side‐by‐side in Figure S13). Each heatmap denotes the growth state of one time point, in this case 0, 2, 4, and 12 h. Bacteria growth is inhibited if they are exposed to a combination of clavulanate and amoxicillin; otherwise, growth is maintained throughout the tested concentration range.

**FIGURE 5:**
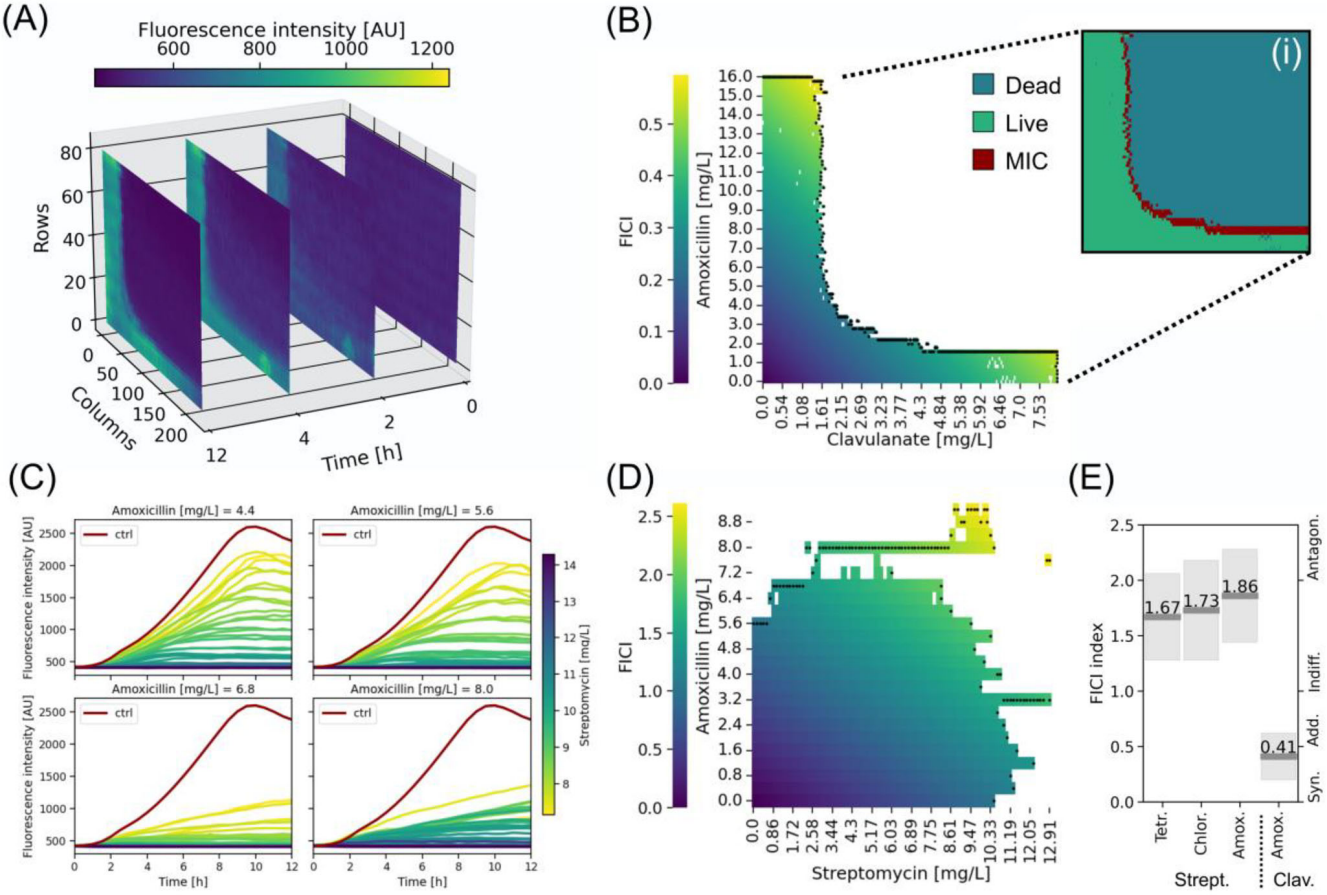
MIC determination on microdroplet arrays and antibiotic cross‐gradient assays. (A) Heatmaps showing the synergistic effect of amoxicillin and clavulanate on an amoxicillin‐resistant *E. coli* strain. The time points 0, 2, 4, and 12 h show the increase in fluorescence intensities (bacteria growth) across the microdroplet array (n = 18 144). The assay layout is according to Figure 1B. The same cross‐gradient assays were further performed with tetracycline, chloramphenicol, and amoxicillin in combination with streptomycin on a susceptible *E. coli* strain. (B) Heatmap of the fractional inhibitory concentration index (FICI) for all droplets, where growth was detected. (i) The insert depicts the classification of the bacteria into dead and live cells, based on the fluorescence signal. Droplets with intermediate states at the minimum inhibitory concentration are designated with “MIC”. (C) Bacterial growth curves of the droplets around the expected MIC of Amoxicillin and Streptomycin (n = 120). (D) FICI values for amoxicillin and streptomycin for all droplets, where growth was detected. (E) From all experiments the mean (dark gray line) and standard deviation (depicted in light gray) of all conditions was determined as a measure to categorize the combined antibiotic effects (Strept.: streptomycin, Clav.: clavulanic acid. Categories are synergistic (Syn.), additive (Add.), indifferent (Indiff.) and antagonistic (Antagon.).

Based on the same method as described in the previous section, we could determine the final growth state by comparison to the control. By setting a threshold, we could then separate the bacteria into three states: live (green), dead (blue), and MIC (red) (Figure 5B‐i). The MIC in red was determined based on the interface between the live and dead region. The FICI values were then calculated according to the following procedure.

The MIC at concentration 0 mg/L for each antibiotic was set to be the MIC alone (MIC_A_, MIC_B_), and all other MICs are related to combinatorial effects (MIC_AB_, MIC_BA_). Finally, the FICI could be determined for every combination with the following equation:

(1) $$FICI=\frac{MIC_{AB}}{MIC_{A}}+\frac{MIC_{BA}}{MIC_{B}}$$

All FICI values are represented in Figure 5B. Synergistic FICI values of <0.5 at the live/dead interface (MIC in red in Figure 5B‐i) were reached with combinations of 1.5–2.5 mg/L clavulanate and 2.5–4 mg/L amoxicillin.

While we selected the amoxicillin‐resistant strain to demonstrate the effect of clavulanate, the susceptible strain *E. coli* strain (ATCC 25922) was used to investigate general combinatorial antibiotic effects with the following antibiotic patterns: tetracycline (0 to 8 mg/L), chloramphenicol (0 to 16 mg/L), and amoxicillin (0 to 32 mg/L) over the rows, each in combination with streptomycin (0 to 8 mg/L) over the columns. The bacterial growth inside the droplets of the amoxicillin/streptomycin combination around the expected MIC are depicted in Figure 5C and the FICI classification in Figure 5D. All further combinations are shown in Figure S14. FICI values are given for all droplets where cell growth is detected.

From every experiment, the mean and standard deviation of all fractional inhibitory concentration conditions were determined as a measure to categorize the combined antibiotic effects. Based on the growth pattern of *E. coli* and the corresponding FICI values of 1.67 ± 0.39 for tetracycline/streptomycin, 1.73 ± 0.45 for chloramphenicol/streptomycin, and 1.86 ± 0.42 for amoxicillin/streptomycin, we found that all antibiotic combinations exhibited an indifferent effect, with a tendency towards antagonism. The combinatorial effects of streptomycin/tetracycline and streptomycin/chloramphenicol are in close alignment with the reported literature [36]. As expected, the amoxicillin/clavulanate combination has an overall smaller FICI value of 0.41±0.11, suggesting a synergistic effect. It should be noted that the determination of MIC_A_ and MIC_B_ alone was not possible, since the highest tested concentrations were not sufficient to alter the growth of the amoxicillin‐resistant bacteria. Therefore, we used 32 and 16 mg/L for the respective MIC_A_ and MIC_B_ in the FICI calculation, which is the break‐point for resistance [37] of the *E. coli* strain ATCC 35218 and the corresponding clavulanate ratio. Further, plate reader measurements confirmed concentration‐dependent growth inhibition of *E. coli* ATCC 35218 under increasing amoxicillin concentrations, which was enhanced in the presence of clavulanic acid. Notably, the strain remained resistant to amoxicillin up to 200 mg/L and to clavulanic acid at concentrations exceeding 31.25 mg/L when applied individually (Figure S15). Wherever applicable, antibiotic concentration ranges were selected based on EUCAST reference values to ensure consistency with established susceptibility testing standards.

### 2.6 Lipid Profiling With MALDI‐MS

After the time‐lapse imaging to monitor the growth of bacteria, we performed an untargeted MALDI‐MS analysis directly on the cross‐gradient assay with the amoxicillin/streptomycin combination. Without any purification steps or sample preparation (except sublimation of the DHB matrix), we retrieved for every droplet on the 20 832 microdroplet array a mass spectrum from 600 to 3000 m/z. We classified the corresponding experiment as before (Figures 5B‐i and Figure S16) into live, dead and MIC categories to perform a principal component analysis on the full microdroplet array including up to 85 mass peaks per droplet (Figure 6A). Apart from the two main clusters (live, dead) we could identify one cluster that resembles the MIC region. In order to rank the most important mass peaks that have an influence on our three categories in an unbiased manner, we applied the random forest classifier. Instead of feeding training data, we used the learning algorithm on a random subset of data to rank all mass peaks based on their importance score. Figure 6B shows the ranking of the 10 most important mass peaks. We found the majority of peaks between 600 and 1100 m/z (Figure S17A) and therefore performed a glycerophospholipid search via the lipid database LIPID MAPS on our top‐10 mass peaks (Figure S17D) [38]. The majority of lipids we found in our MALDI‐MS analysis are phosphatidylethanolamines (PE) and Phosphatidylglycerol (PG), which comprise about 75% and 20%, respectively of all lipids in *E. coli* and align with the reported literature [39, 40, 41, 42]. The most influential mass peaks at 608.316 and 692.647 m/z were the phosphatidylethanolamines PE26:0 and PE32:0, respectively. By comparing the signal intensities of PE26:0 to the GFP fluorescence intensities of the last time point across the microdroplet array, we find it as an ideal marker to determine the bacterial growth of the tested strain in a label‐free manner (Figure 6C, side‐by‐side in Figures S18 and S17B). MALDI‐MS analysis enabled extraction of MIC values and corresponding FICI indices, yielding an average FICI of 2.21 ± 0.73. This value falls within the same interaction categories (indifferent towards antagonistic) and agrees well with fluorescence‐based measurements, supporting the consistency between both readout modalities (Figure S19).

**FIGURE 6:**
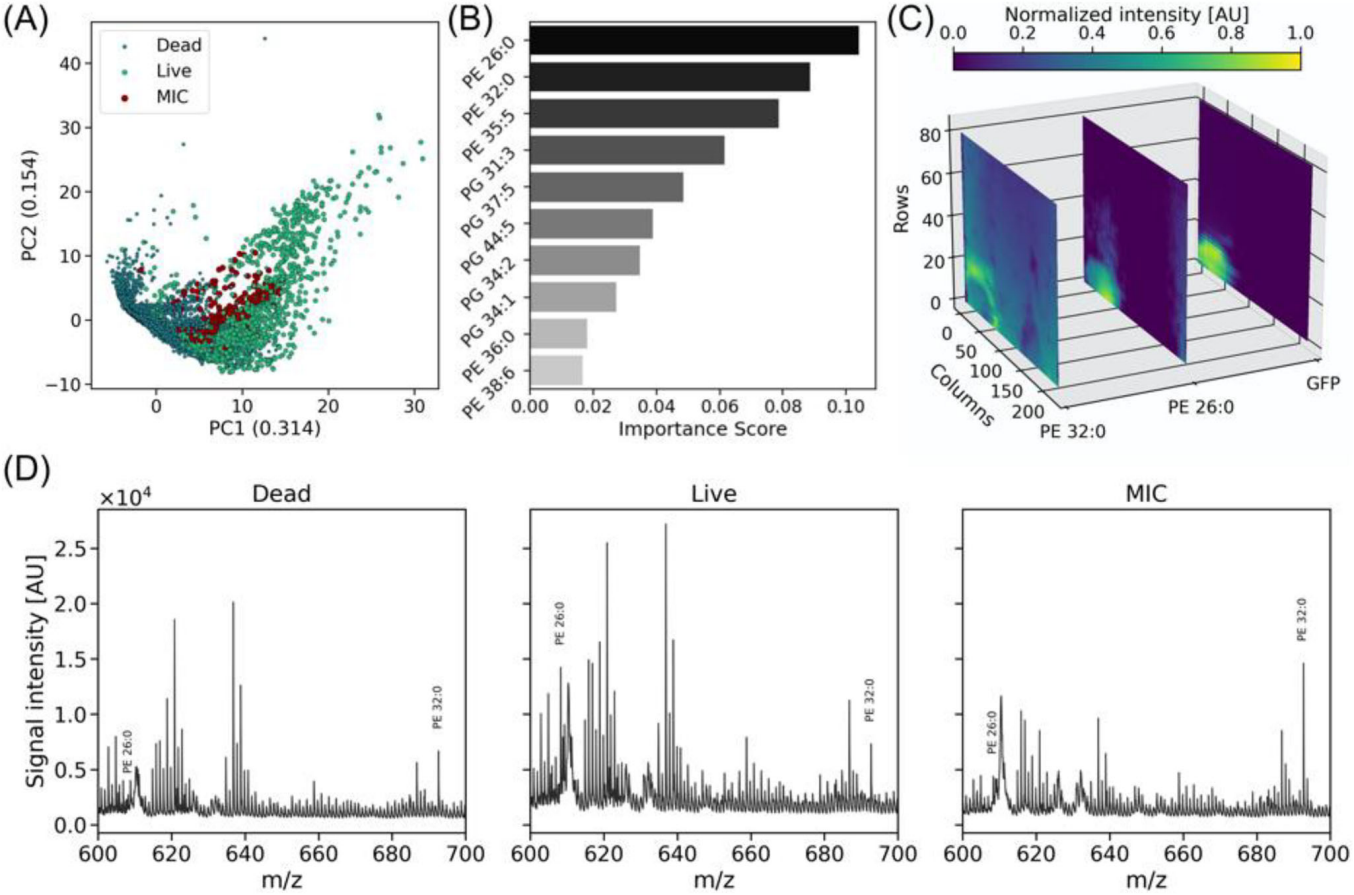
MALDI‐MS analysis of *E. coli* under amoxicillin/streptomycin treatment. (A) Principal component analysis of all mass peaks (n = 85) of every droplet on the microdroplet array (n = 20 832). Dead, live, and MIC are categorized based on the fluorescence heatmap (Figure S16). (B) Random forest classification was applied as a learning algorithm to detect the most important mass peaks. (C) The mass peaks 608.316 and 692.647 m/z with the highest importance score matched the two lipids PE 26:0 and PE 32:0, respectively. The intensity of both mass peaks across the droplet microarray and the GFP signal of the last time point obtained by the fluorescence measurement are resembled for comparison in three heatmaps. (D) Mass spectra of the three classified regions, "Dead", "Live", and "MIC", with annotated peaks for the lipids PE 26:0 and PE 32:0.

Interestingly, the lipid PE32:0 with the second highest importance score shows an increase in signal intensity in the MIC region (Figure 6C,D and Figure S17C). It is known that *E. coli* undergoes morphological changes when exposed to drugs, especially around the MIC. The effect of drugs on the lipid profile are less studied, but has been reported e.g. for the antibiotic compound Naringenin [43]. Further, it was reported that cells undergo a shift in the lipid acyl chain length when entering the stationary growth phase, which is connected to e.g. lack of nutrients or oxygen [44]. A similar increase in acyl chain length was observed by comparing susceptible and resistance bacteria strains [45]. Treatment of *E. coli* with ciprofloxacin increased lipid diffusivity and elongation [46, 47]. Overall, the literature suggests that bacterial lipid composition is significantly influenced by stress responses, a trend that is also evident in our experiment. These findings could help clarify how bacteria alter their membranes to resist specific antibiotics [43, 47] and identify distinct lipid biosynthetic pathways that could serve as new drug targets or improve antibiotic efficacy [48, 49]. Given the large number of tests that can be performed on a single platform, machine learning‐based analysis could in the future aid in identifying biomarkers or lipid patterns [50].

## 3 Conclusion

We have introduced a platform to perform high‐throughput assays for combinatorial antibiotic drug testing against *E. coli* on highly dense open microdroplet arrays. Owing to several advantages over conventional approaches, we achieve an unprecedented density of 20 832 cultivation compartments on the area of a microscopy slide, corresponding to more than 50 widely used plates with 384 wells. Due to the multi‐channel printhead that combines different drugs shortly before droplet formation, we can generate complex checkerboard layouts in under 25 min using total volumes of about 25 µL. In contrast, if multiple reagents have to be introduced in conventional liquid handling robots or pico/nanodispenser, the time increases with the number of filling steps. Our unique platform exceeds existing systems not only in regard to throughput but also offers an immense increase in information density. We obtain kinetic information about the bacterial growth via time‐lapse fluorescence microscopy. The small droplet volume and height further enable us to monitor changes in the morphology of the bacteria exposed to different drugs. We investigated here the combinatorial effect of two drugs using a single microdroplet array. In the future, the large droplet arrays could be used more efficiently, for example by integrating multiple checkerboard assays per array (as demonstrated by the nine MIC tests shown in Figure 4, all performed on a single platform), and by systematically introducing a third drug via one of the channels currently used for medium supply. While systematic combinations with even more drugs are also feasible, they would require either a modified printhead with more supply channels, or sequential filling of droplets across several spotting runs.

Final untargeted MALDI‐MS analysis gives insights into hundreds of lipids and potentially other biomolecules in the future. In our findings, the lipids with the highest importance scores from the microscopy‐supported MALDI‐MS data led to the identification of two lipids that underline altered lipid composition in response to antibiotic stress and could serve as excellent biomass markers for label‐free MIC determination by MALDI‐MS for the tested strain, while validation across other pathogens is needed to confirm generalizability.

Since drug interaction studies involve phenotypic screening with e.g., microscopy, while the identification of resistance mechanisms relies on methods such as MALDI‐MS, our platform can combine both areas of research. This is of importance to address the need for new innovative solutions to combat antibiotic resistance by enabling the development of effective and sustainable therapies. In particular, when multimodality is desired, our platform combines the capability to monitor cell growth and morphology through microscopy with the possibility to elucidate chemical structures via MALDI‐MS. Furthermore, our platform can be employed to investigate other combinations of drugs and bioactive compounds as well as other cell types and co‐cultures.

## 4 Experimental Section

### 4.1 Experimental Setup

The hydrophobic/hydrophilic microarray plate that contains the droplet hosting sites was prepared in the cleanroom. The fabrication process and experimental setup is described in our previous publication [26]. Here, we used a “printhead” instead of a single capillary to deliver fluids to the plate. This printhead holds five capillaries, each is connected to another fluid reservoir. In our previous study, the printhead was characterized and discussed in more detail [27]. The platform is modular and scalable, and previous work demonstrated mixing of up to five independent compounds using individually controlled pressure channels. In principle, the number of drugs can be further increased by reducing buffer channels, increasing the number of fluid inlets, performing sequential deposition steps, or pre‐mixing selected drug combinations.

### 4.2 Bacteria Cultivation

Bacterial strains [46] were preserved as cryo‐stocks with 25% glycerol at ‐80°C. Before each experiment, 100 µL of the stock culture was inoculated into 3 mL of cation‐adjusted MHB II, supplemented with antibiotics as needed for plasmid maintenance: ATCC25922 [pSEVA271_sfgfp] with 50 µg/mL kanamycin sulfate, and ATCC35218 [pSEVA271_sfgfp] with 50 µg/mL kanamycin and 100 µg/mL amoxicillin. The cultures were incubated at 37°C in a shaking incubator (Minitron, Infors HT) at 200 rpm. Once the OD_600_ reached 2–4, the cultures were diluted to an OD_600_ of 0.01 or 0.25 (depending on the experiment) in fresh cultivation medium. The diluted cultures were used immediately for droplet generation.

### 4.3 Reagents

Suppliers and catalogue numbers for all reagents are listed in the supplementary Table S1. Before droplet formation, the microarray plate was covered with 3 mL of HFE‐7500 oil (3 m, USA). The fluorescent dyes fluorescein (GFP channel), sulforhodamine B (mCherry channel), dextran Cascade Blue (DAPI channel), and dextran Alexa Fluor 647 (Cy5 channel) were dissolved in purified water at concentrations of 10 µm. All reagents were transferred to 1.5 mL Eppendorf tubes prior to use for the experiments. For the drug tests, we used the concentrations as listed in Figure S9. The stock solution of all samples was prepared in cation‐adjusted MHB II supplemented with 50 µg/mL kanamycin sulfate. All channels contained aqueous solutions with comparable viscosities (MHB or water‐based solutions). Due to the low concentrations of antibiotics and fluorescent dyes, no measurable viscosity differences were expected between channels.

### 4.4 Fluorescence Imaging and Analysis

For fluorescence measurements, the oil‐tight tray including the generated microdroplet array was transferred to a Ti2 Eclipse (Nikon, Japan) with a SOLA SE II (Lumencor, USA) light source and a DIQ2 camera (Nikon, Japan). To mitigate evaporation, small water trays are placed inside the oil bath, and the tray is sealed with adhesive breathable PCR plate foil. The objective used for all measurements was a CFI Plan Apochromat Lambda 4x (Nikon, Japan). The intensity and exposure time were set to 200 ms and 20% intensity for all channels. For the bacterial experiments, we reduced the exposure time to 50 ms.

We used filter sets for GFP, mCherry, DAPI, and Cy5 from Nikon. Image analysis was performed using a custom‐built MATLAB script by extracting the mean fluorescence intensity values of the spots. The area of interest was chosen to be slightly smaller than the spot diameter to avoid misrepresentation of fluorescence values due to the appearance of a halo around some droplets.

### 4.5 MALDI Procedure and Analysis

100 mg 2,5‐DHB matrix (Sigma–Aldrich, USA) dissolved in acetone was added to the bottom of a custom‐made sublimation chamber [51]. The plate was attached to the bottom of a cold finger and inserted in the chamber. The chamber was evacuated to a pressure of 0.2 mbar using a rotary vane pump. Then, an ice slush was filled into the cold finger, and thermal stabilization of the system was allowed for 3 min. Afterward, a copper heating bath was used to heat the chamber to 140°C for 15 min to sublime the DHB matrix. Analysis was carried out with a RapifleX from Bruker Daltonics, USA. In positive reflector TOF/TOF mode, 1000 shots per spot were collected at a repetition rate of 10 kHz with a fixed laser power of 90%. A mass range of 600–3000 m/z was chosen with a digitization rate of 1.25 GS/s. Spectra were accumulated by the FlexControl software and exported using FlexAnalysis Batch Process software. Peak identification was performed beforehand with the FlexAnalysis software, and further data analysis was carried out in Python. MALDI‐MS measurements were acquired at an average rate of ∼0.24 s per spot (∼4.2 Hz), resulting in a total acquisition time of approximately 1.4 h for a full 20 832‐droplet array.

### 4.6 Statistical Analysis

Droplet evaporation was assessed by comparing droplet volumes at 0 and 12 h using a two‐sided t‐test with a significance threshold of α = 0.05. Growth across culture formats was compared based on calculated doubling times, with differences between groups assessed using two‐sided Welch's t‐tests and α = 0.05. For the time‐resolved antibiotic response analysis, the deviation time point was defined as the earliest time point at which fluorescence intensity differed by at least 5% from the corresponding control condition. Unless otherwise stated, data are reported as mean ± standard deviation, and n indicates the number of droplets or bulk culture replicates analyzed.

## Author Contributions

P.S.D. and M.B. designed the study. M.B. developed the method, planned and performed the experiments, and processed and analyzed the experimental data. C.L.D. integrated the force sensor and provided the script for analyzing the fluorescence images. F‐L.B. helped design the biological studies. I.O. performed and analyzed the plate reader and shaker flask experiments. L.B. assisted with the MALDI‐MS analysis and lipid profiling. P.S.D. supervised the study. M.B. and P.S.D. wrote the manuscript that all authors approved.

## Conflicts of Interest

The underlying technology is part of a patent filed by M.B., C.L.D. and P.S.D. Application number EP24200716.9.

## Supporting information




**Supporting File**: advs76678‐sup‐0001‐SuppMat.pdf.

## Data Availability

Data, mass spectra, and Python codes are available at https://doi.org/10.5281/zenodo.20845305. Microscopy images are available from the authors upon request.
