# Supplementary material for: High‐Density E. Coli Cultivation Arrays for Combinatorial Drug Testing With Subsequent Lipid Profiling by MALDI‐MS
Source: Adv Sci (Weinh). 2026 Jul 23;13(57):e76678. doi: 10.1002/advs.76678 (PMC13395391; doi:10.1002/advs.76678)
Supplement: Supplementary file 1 [file ADVS-13-e76678-s001.pdf]

Supplementary Information

High-density *E. coli* cultivation arrays for combinatorial drug testing with subsequent lipid profiling by MALDI-MS

*M. Breitfeld, C. L. Dietsche, F-L. Born, I. Onipko, L. Blaase and P. S. Dittrich**

Department of Biosystems Science and Engineering, ETH Zürich, Schanzenstrasse 48, CH-4056 Basel, Switzerland

*corresponding author:
Petra S. Dittrich
ETH Zürich
Department of Biosystems Science and Engineering
Schanzenstrasse 44
CH-4056 Basel (Switzerland)

e-mail: petra.dittrich@bsse.ethz.ch

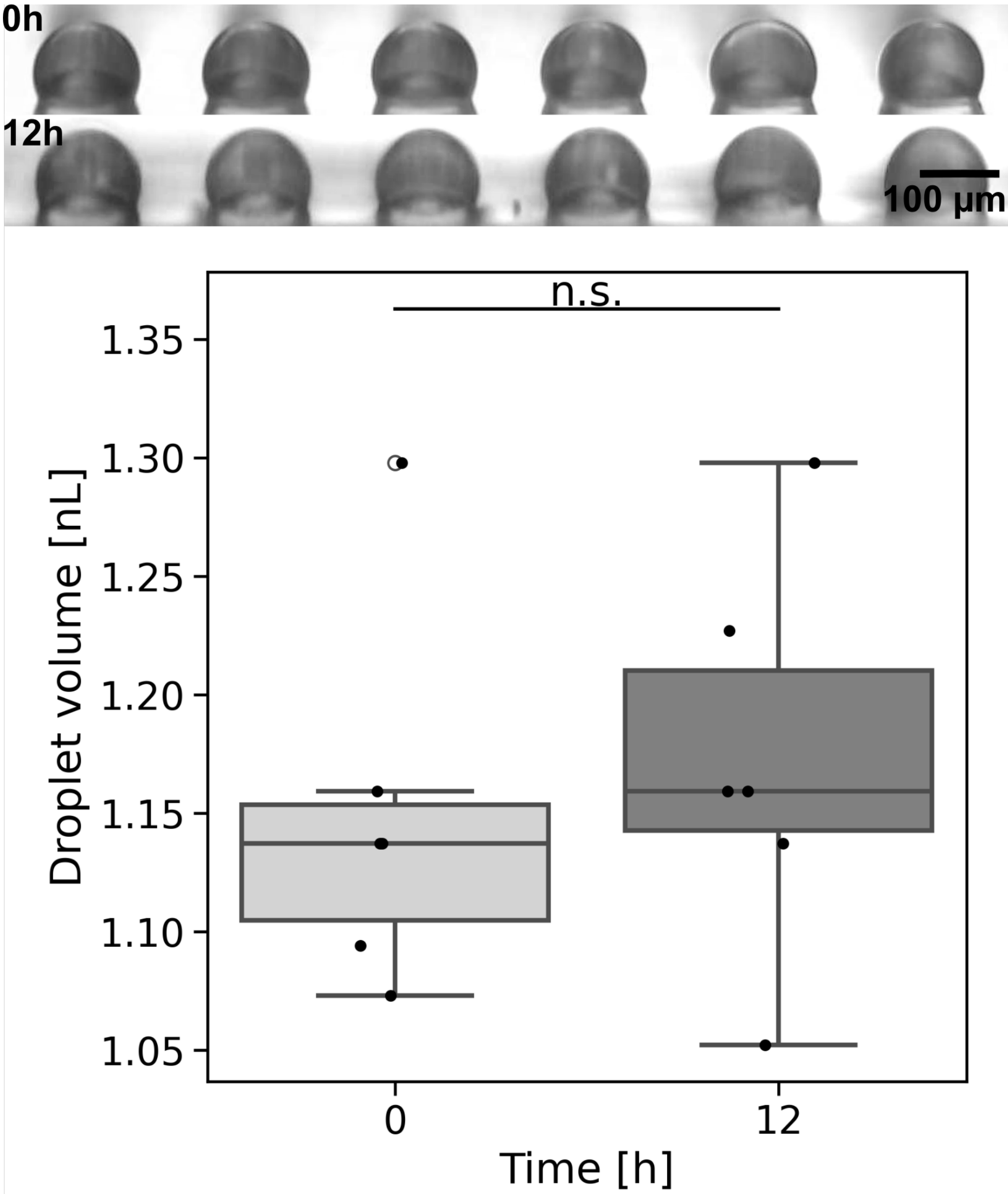

Figure S1. Droplet evaporation was assessed by side-view imaging of n=6 droplets at 0 h and after 12 h, showing no significant change in droplet volume (two-sided t-test, α=0.05).

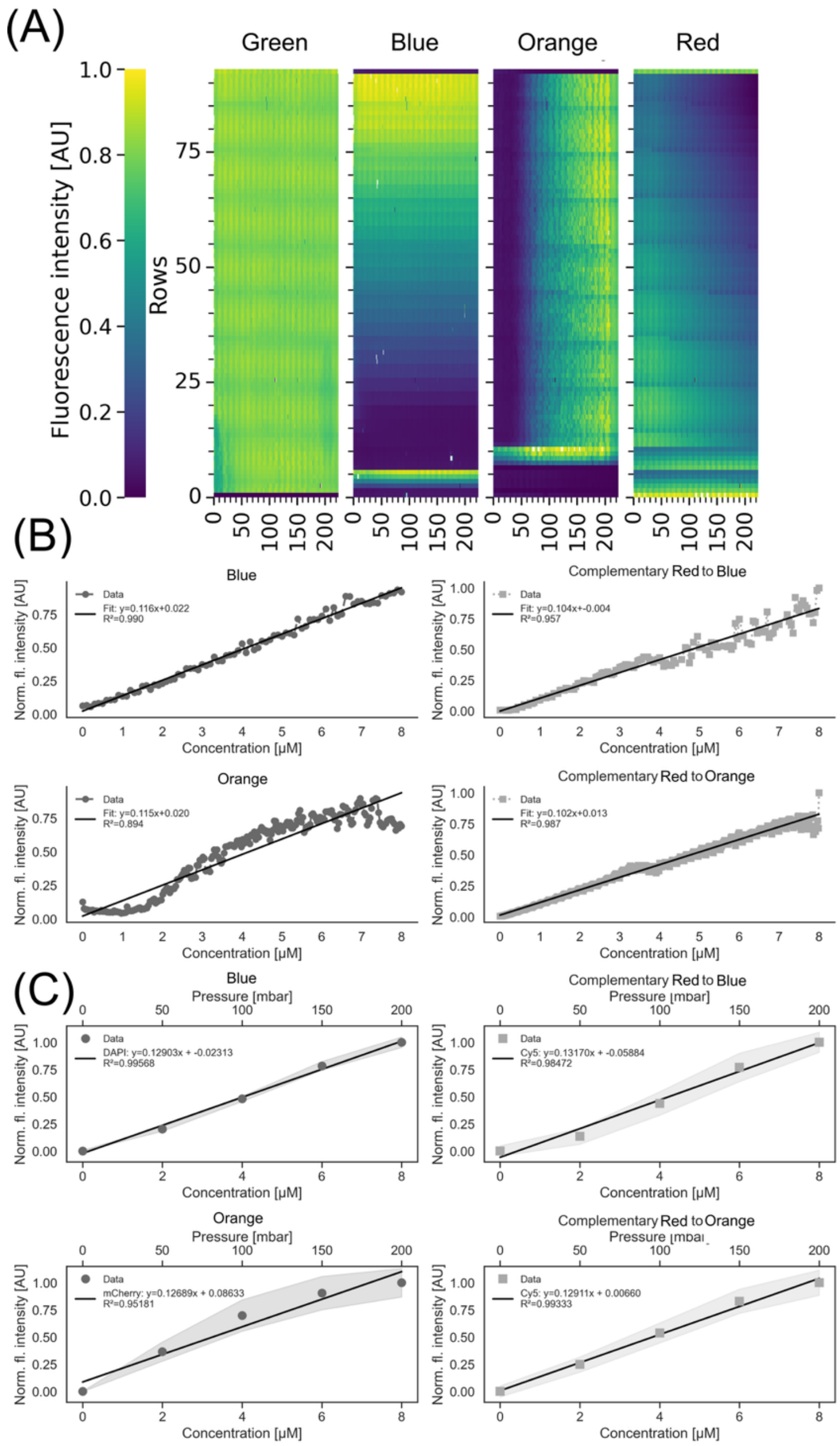

Figure S2. (A) Heatmaps of a 20832-droplet array plate. Droplets contain the four dyes in a determined concentration pattern. Fluorescein is added to all droplets at the same concentration (column "green"). Dextran Cascade Blue is added as in a concentration gradient, here from top to bottom reduced (column "blue"). Sulforhodamine B (SRB, "orange") is increased from left to right, and dextran Alexa Fluor 647 ("red") is used at constant concentration. Control lines without dye (dark blue), or high dye concentrations (yellow) are included in the pattern as well. Note: The images are raw data. The visible square pattern is due to the imaging procedure. Subsets of droplets are imaged at the same time and the images are stitched together afterwards. Intensity deviations are visible at the interfaces of the images. (B) Fluorescence-concentration calibration curves for each dye. Dextran Cascade Blue and its complementary dextran Alexa Fluor 647 signals are derived from droplets located in the first and last columns, while SRB and its complementary dextran Alexa Fluor 647 signal are derived from droplets in the first and last rows. This selection ensures that the full dextran Alexa Fluor 647 concentration

gradient is captured in both cases. (C) shows the fluorescence-concentration calibration curves for each dye of the corresponding control rows (rows 2-6 dextran Cascade Blue – dextran Alexa Fluor 647 and rows 7-11 SRB – dextran Alexa Fluor 647), including the applied pressures for each of the five concentration steps (n=224 droplets per condition).

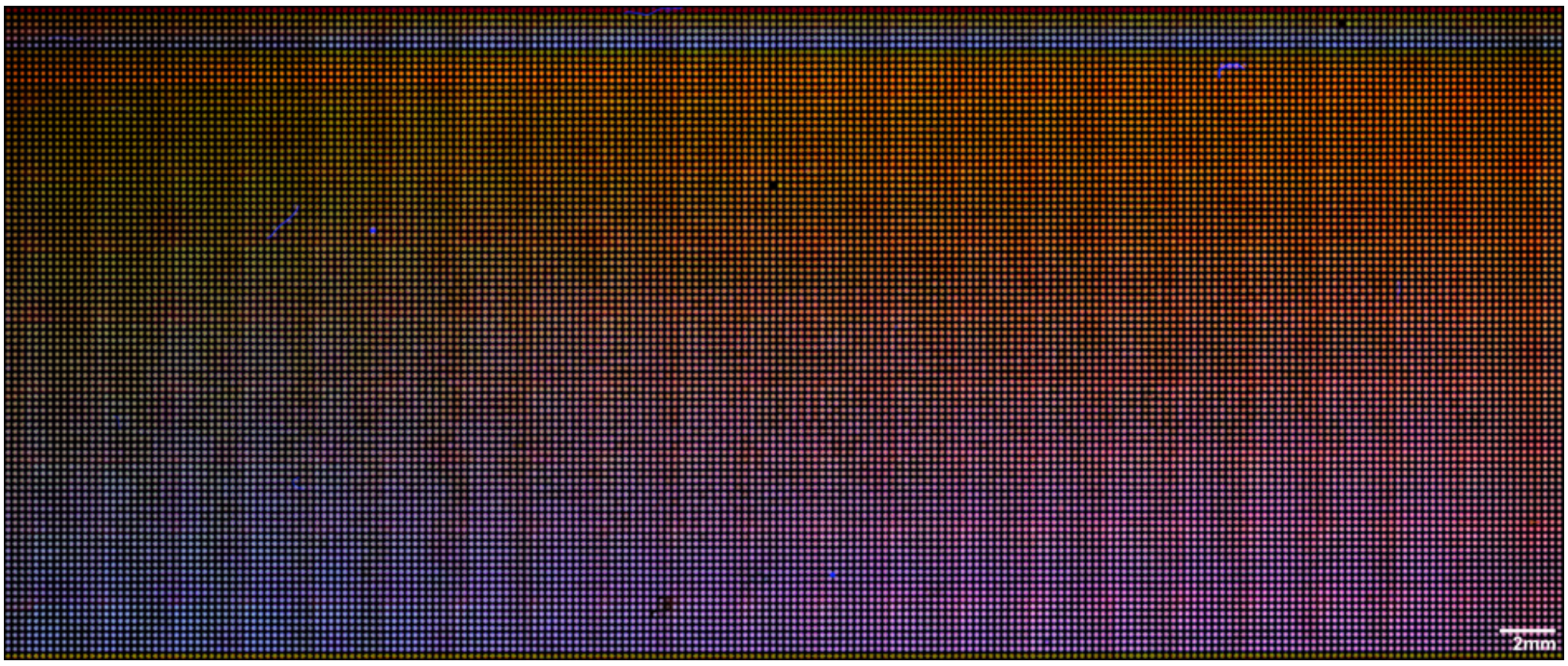

Figure S3. Expanded view of the 20’832-droplet array, all colors overlaid.

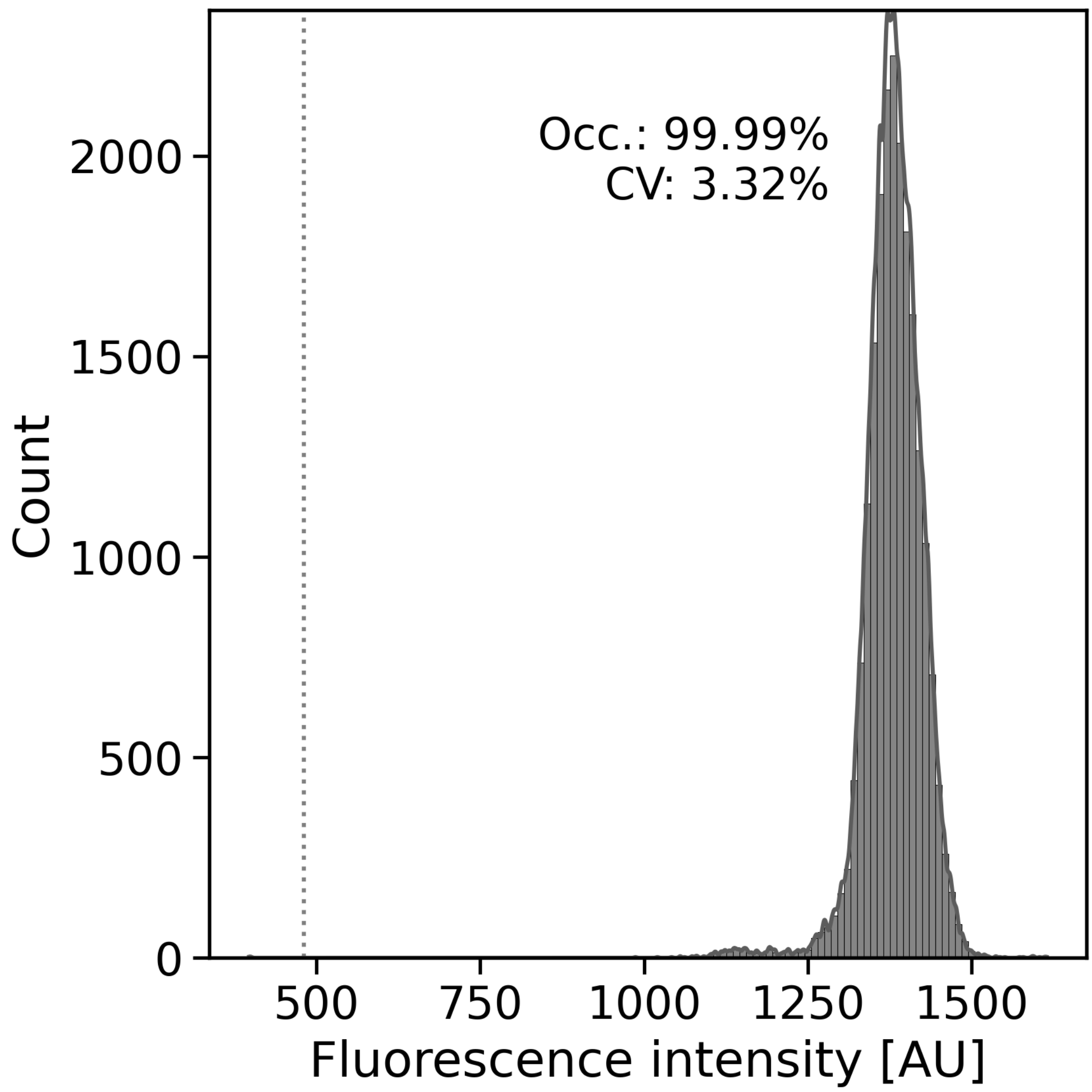

Figure S4. Histogram summarizing the distribution of fluorescein fluorescence intensities across all droplets (n=20’608). The limit of detection (LOD, dashed line) was determined by kernel density estimation of the signal distribution, with the minimum between background and signal populations used as threshold. Occupancy (Occ) was calculated as the percentage of droplets with signal intensities above the LOD and below saturation relative to the total number of droplets, while the coefficient of variation (CV) was calculated as the standard deviation divided by the mean signal intensity and expressed as a percentage.

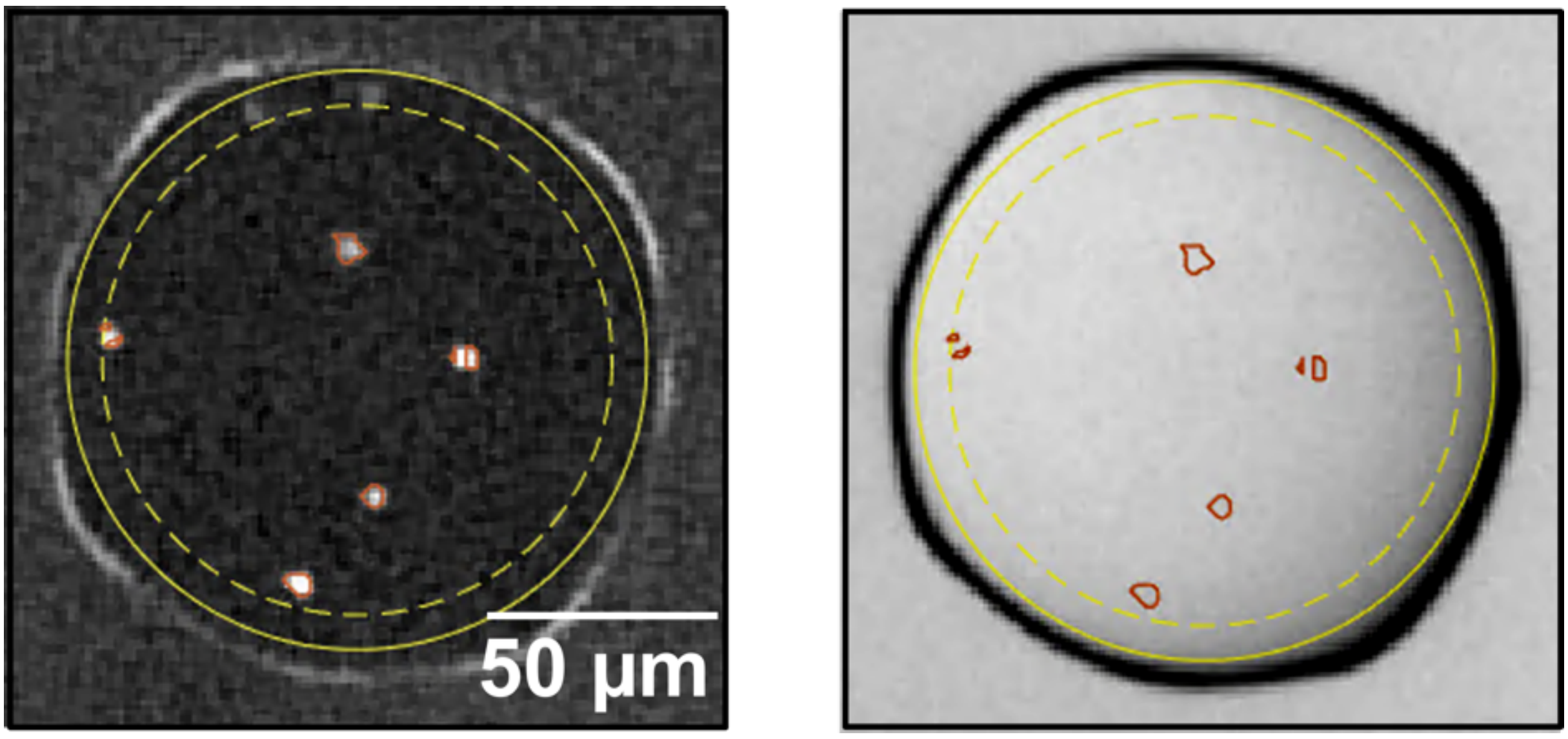

Figure S5. Fluorescence and brightfield images of one droplet, here filled with five bacteria, illustrating the automated image analysis to detect the starting cell number within the droplets at t=0h.

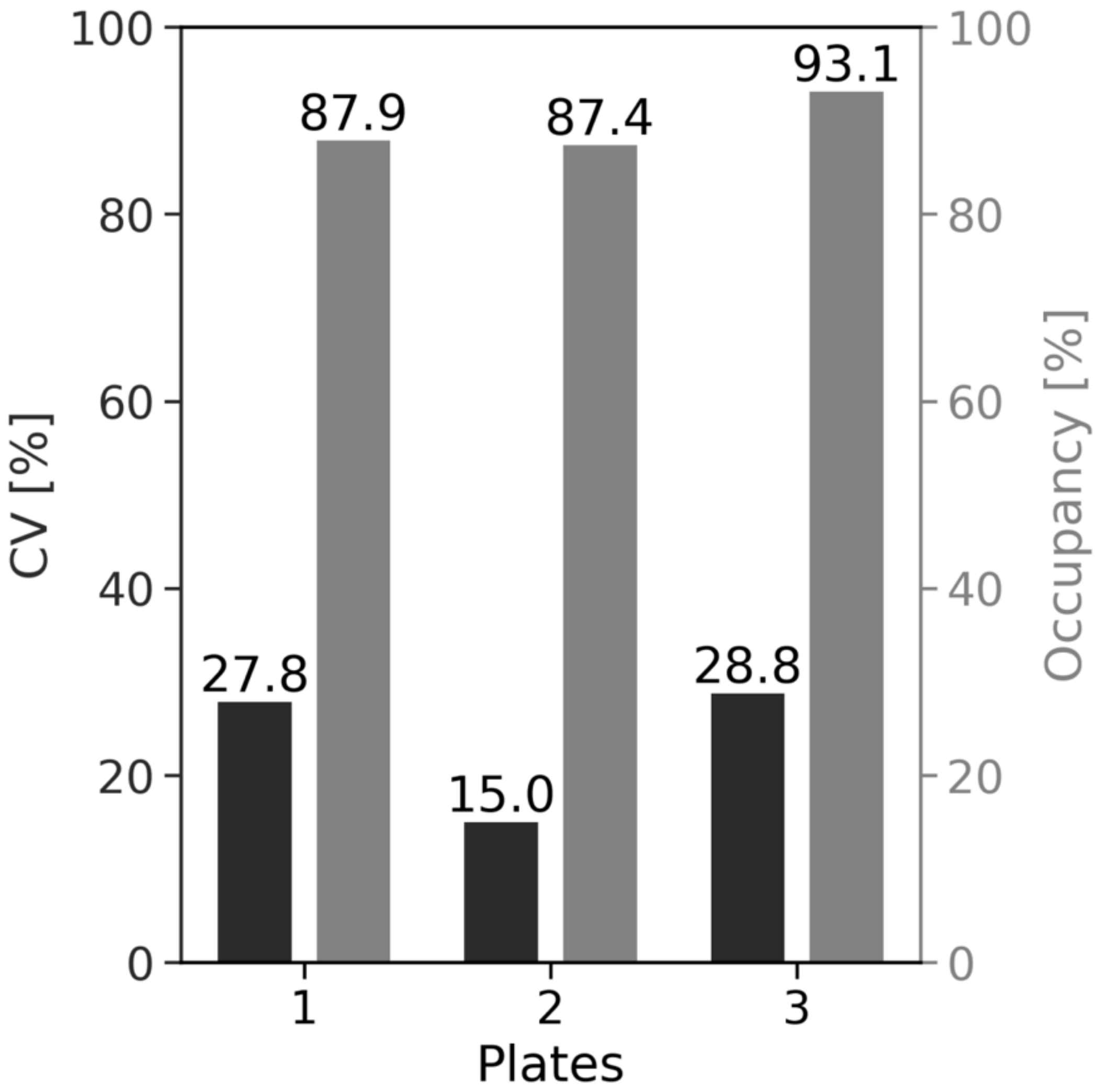

Figure S6. Comparison of three independent experiments on three different plates. Endpoint analysis of the droplet occupancy and growth homogeneity (CV) across the plates.

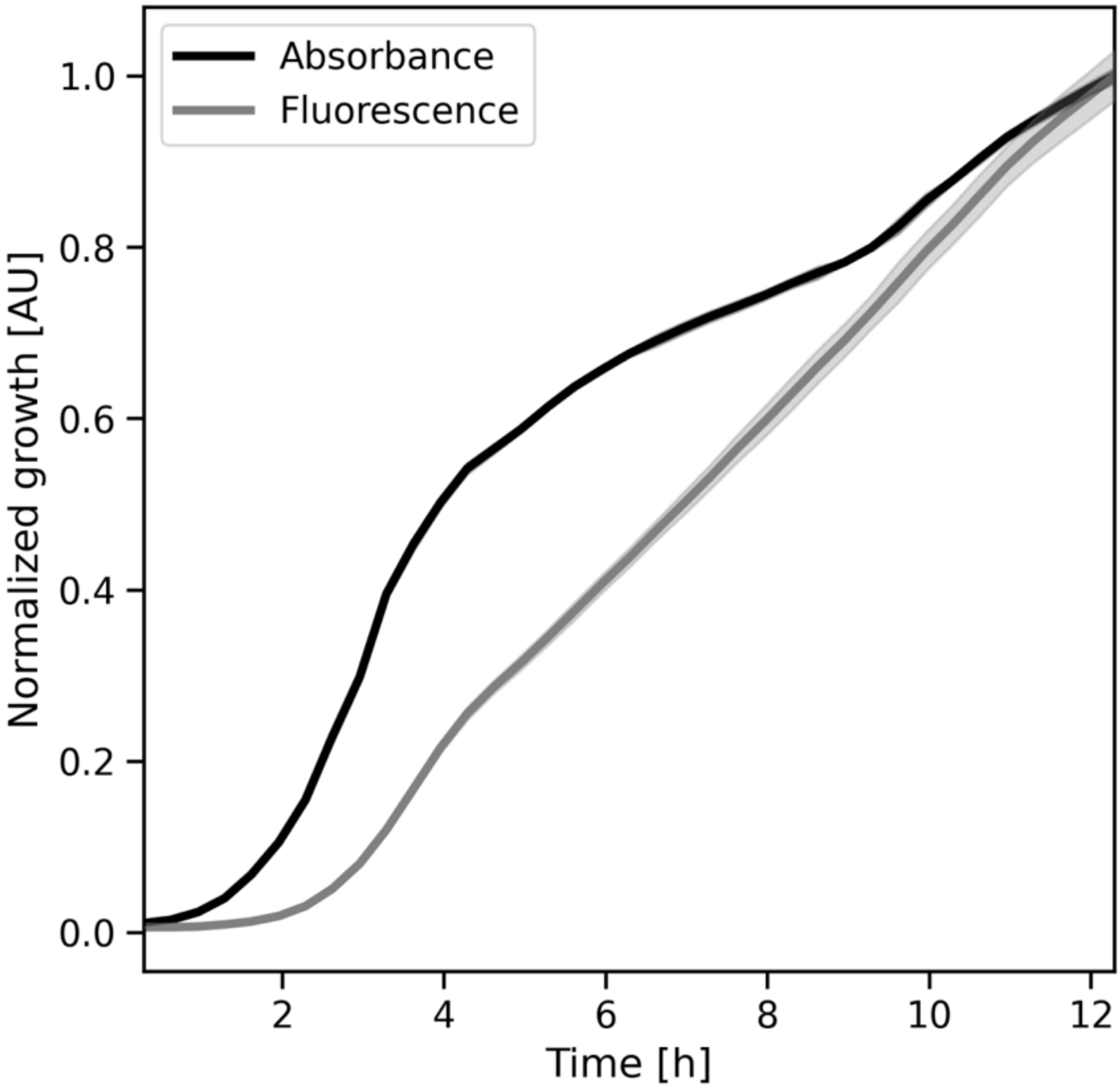

Figure S7. Two growth curves were generated using a plate reader from the same samples, with measurements based on absorbance and fluorescence readouts (n=3). Reference strain *E. coli* ATTC 25922 expressing sfGFP was used for this experiment.

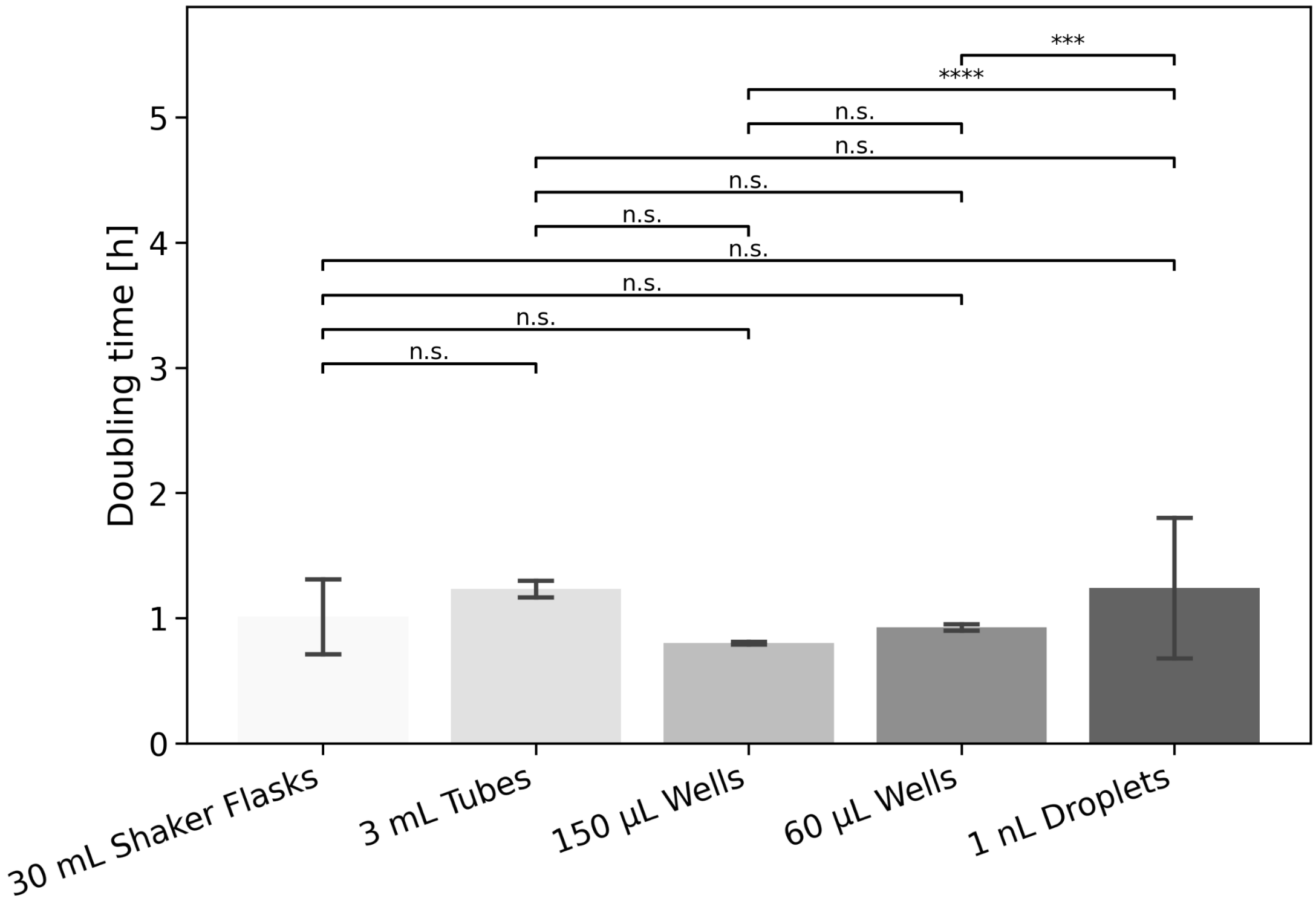

Figure S8. Growth comparison across different culture formats, including droplets (n=50) and bulk conditions (n=3). Doubling times were evaluated using a two-sided Welch's t-test (α=0.05). Significant differences were observed between droplets and well plate formats, while no significant difference was detected between larger-scale cultures (30 mL shaker flasks and 3 mL tubes).

Conc. [mg/L]	Amoxicillin	Ampicillin	Carvenicillin	Chloramphenicol	Ciproflaxacin	Meropenem	Polymixin B	Streptomycin	Tetracyclin
1/4 MIC	1	1	2	1	0.002	0.00575	3	2	0.5
1/2 MIC	2	2	4	2	0.004	0.0115	6	4	1
1 MIC	4	4	8	4	0.008	0.023	12	8	2
2 MIC	8	8	16	8	0.016	0.046	24	16	4
4MIC	16	16	32	16	0.032	0.092	48	32	8

Figure S9. Antibiotics and concentrations for the MIC assay.

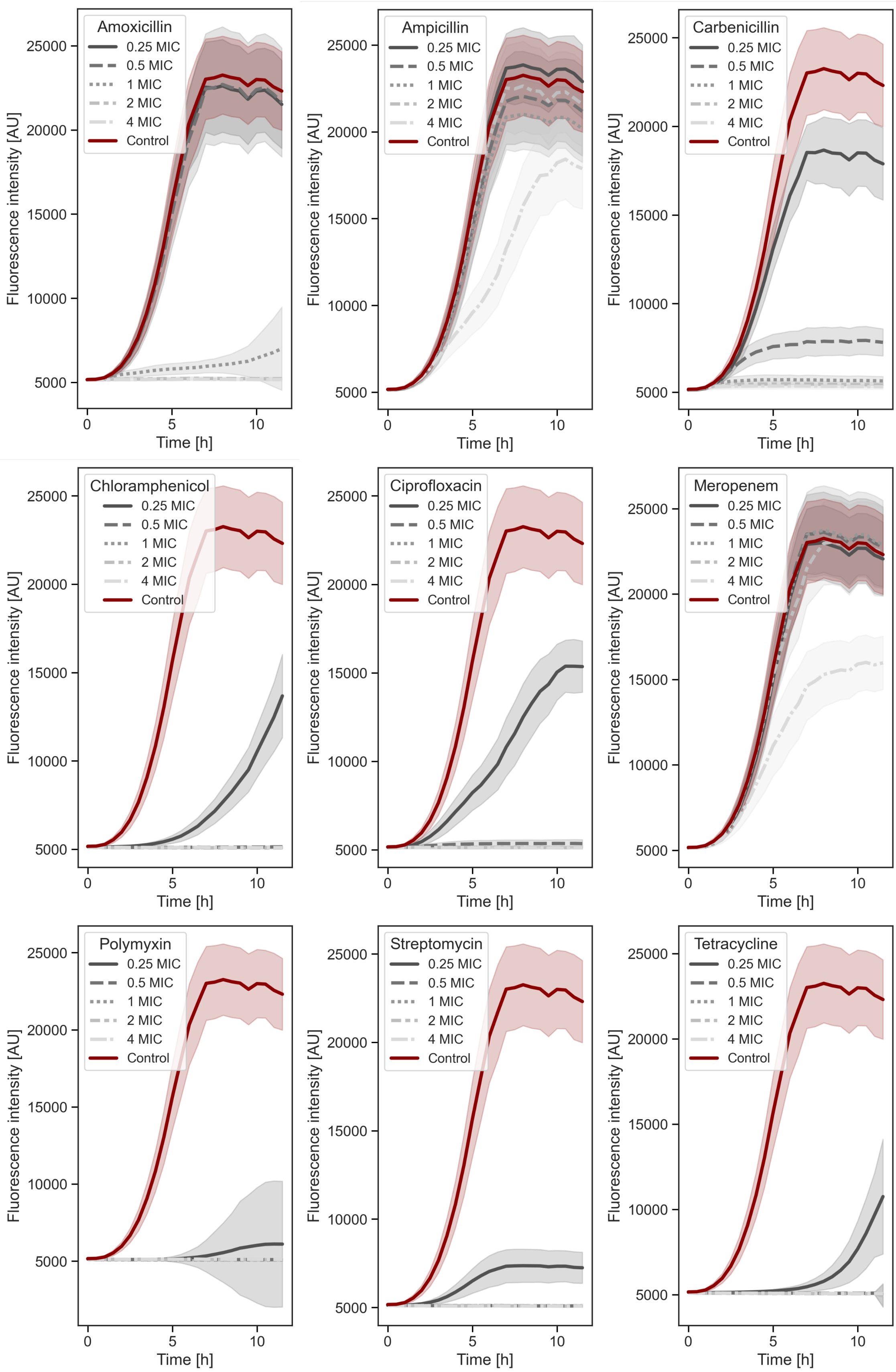

Figure S10. Growth curves of bacteria on the microdroplet array under exposure of various antibiotics and antibiotic concentrations (n=90 per growth curve).

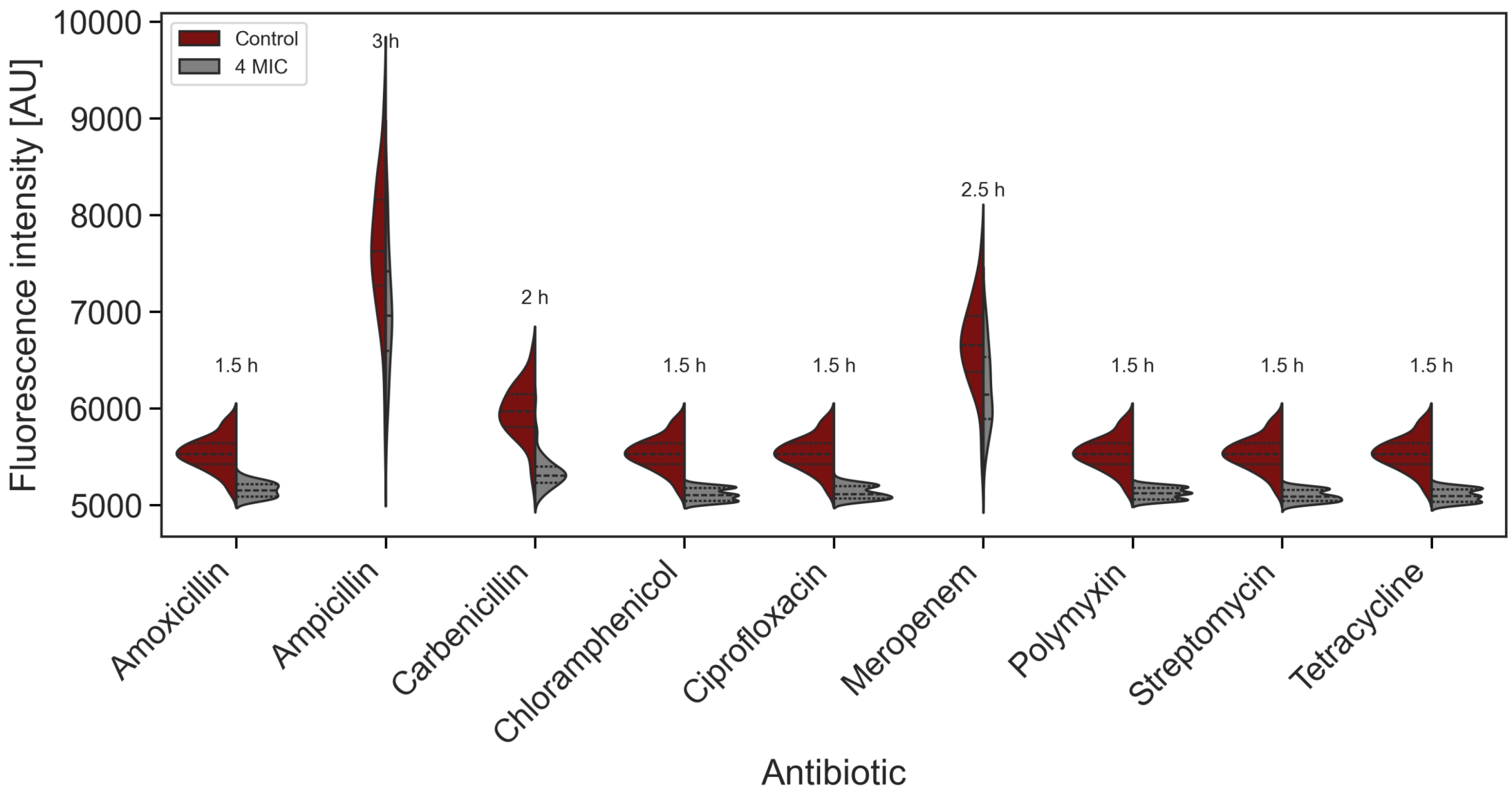

Figure S11. Time-resolved analysis of growth deviation between control and 4xMIC conditions across different antibiotics. Droplets (n=90) were monitored, and the deviation time point was defined as the earliest time at which a 5% difference in fluorescence intensity compared to the control was observed.

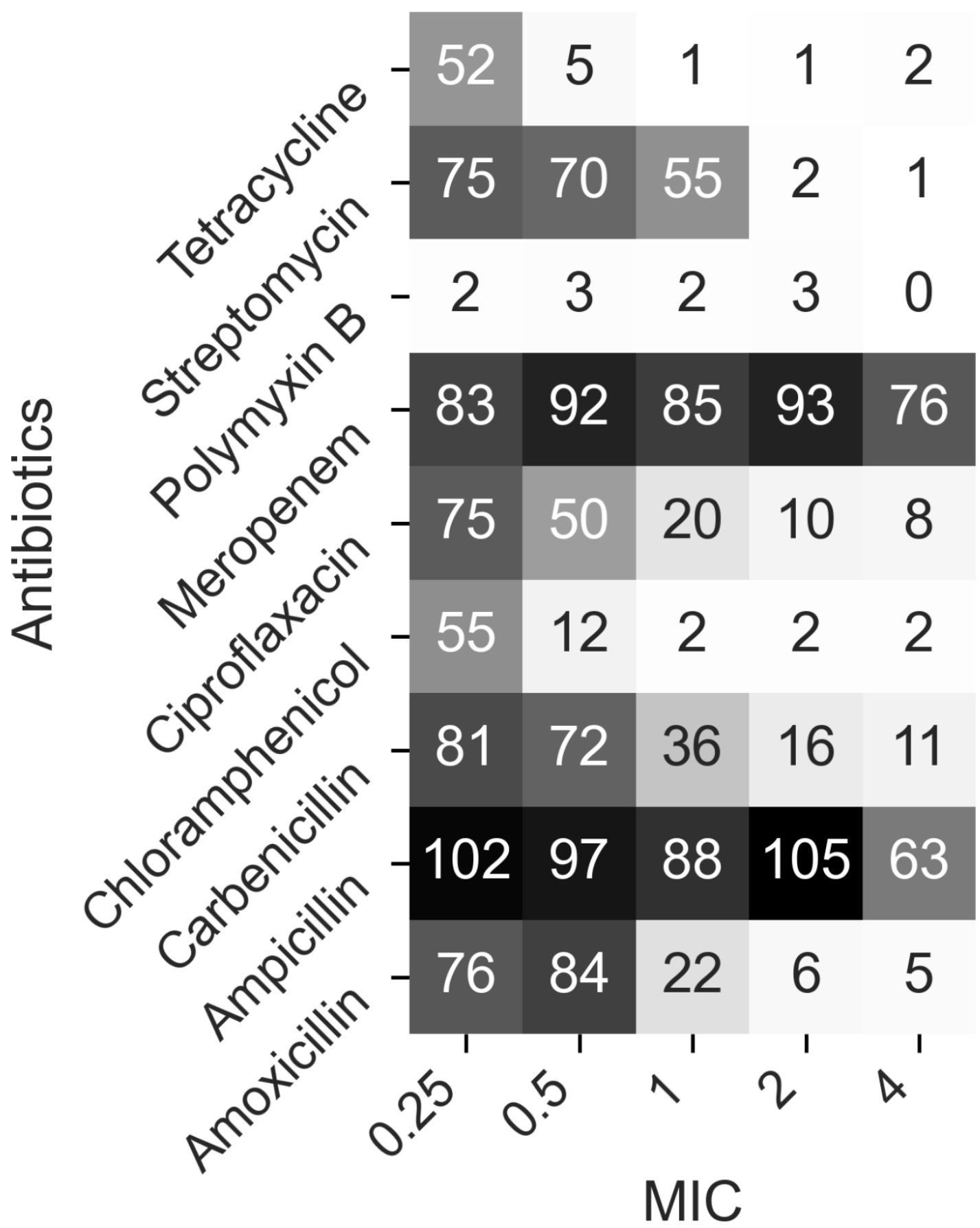

Figure S12. MIC determination after 11 h incubation based on plate reader measurements.

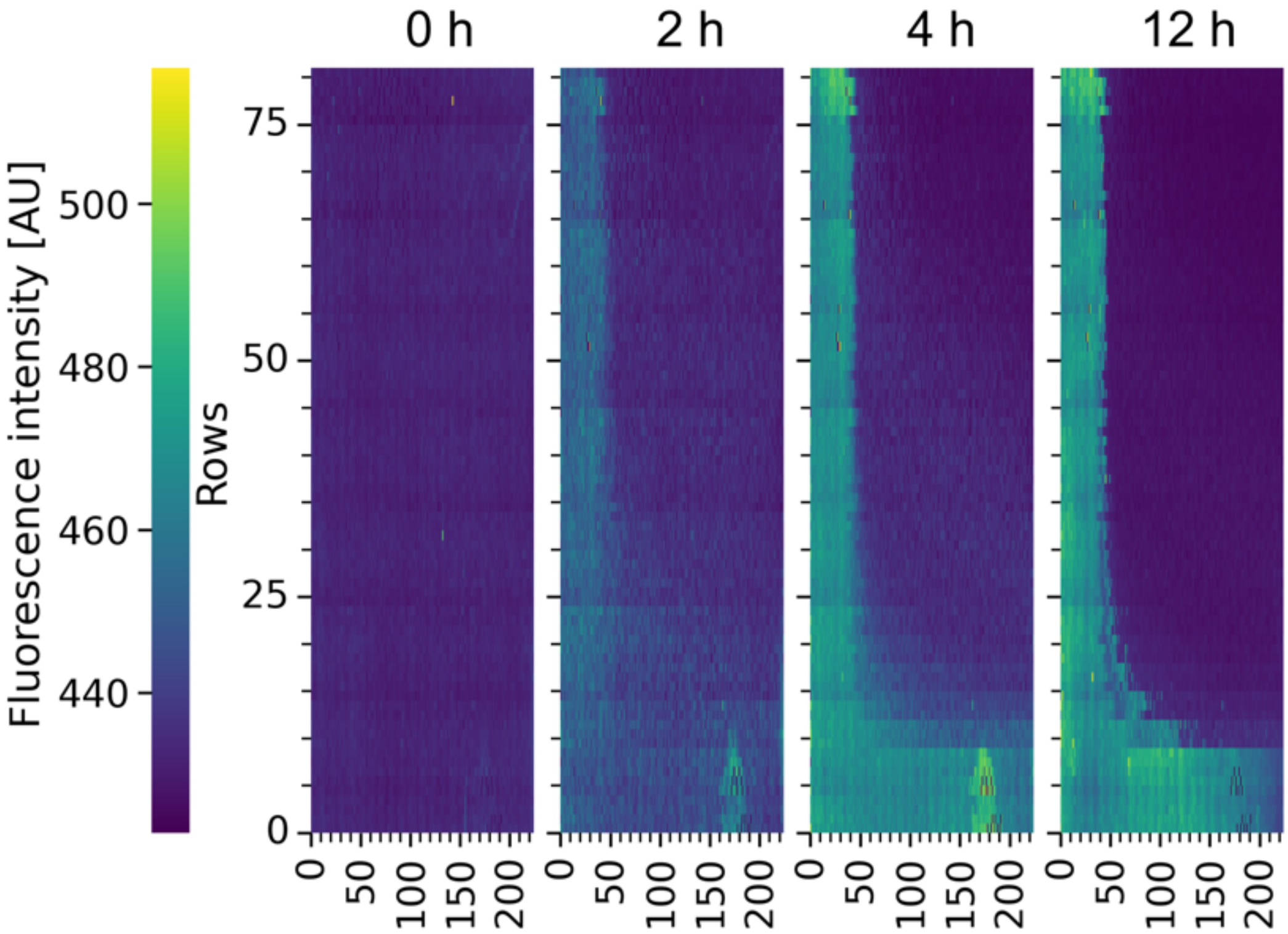

Figure S13. Side-by-side heatmaps for the amoxicillin/clavulanate combination at different time points.

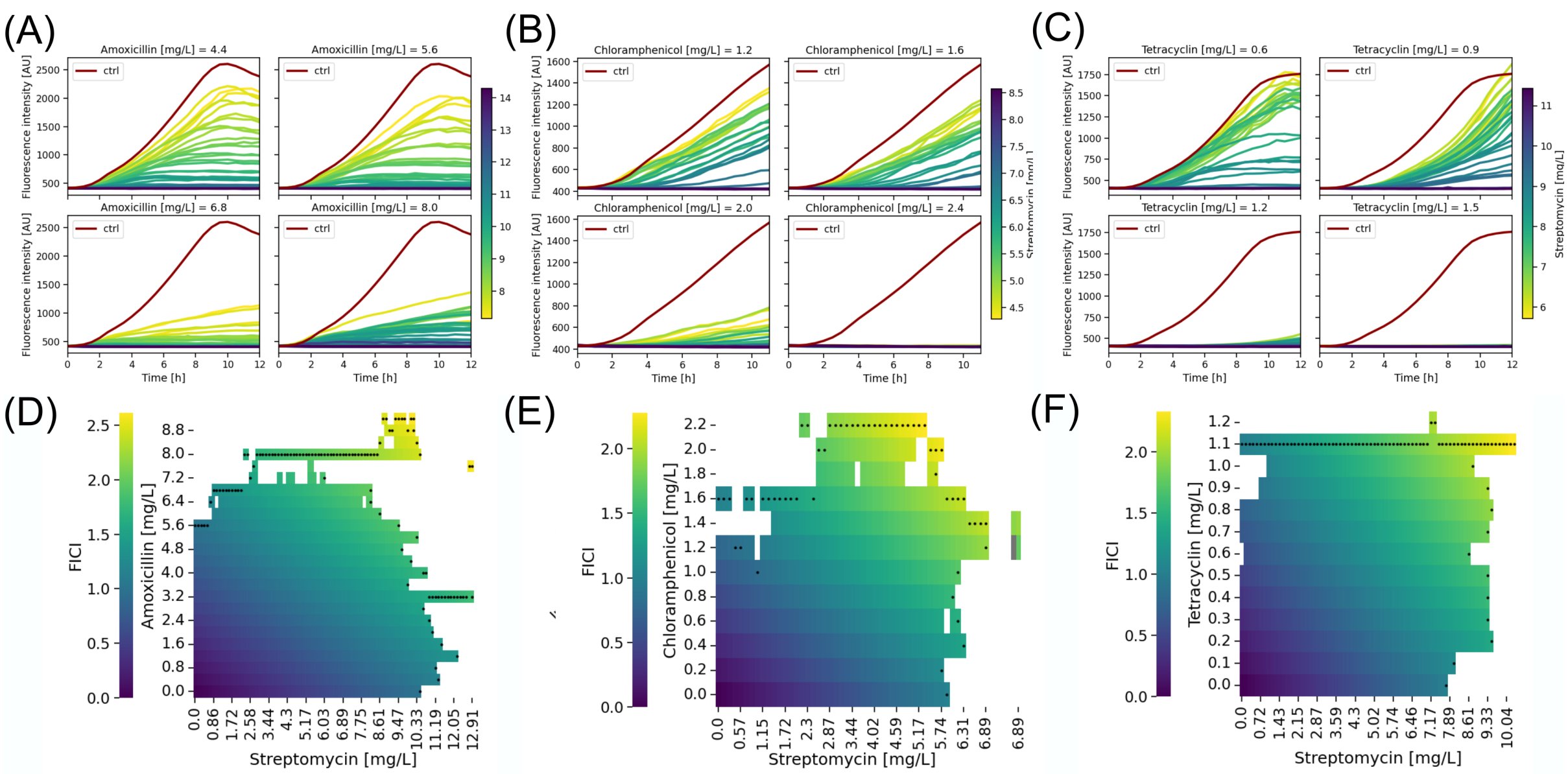

Figure S14. Growth curves and FICI heatmaps of all combinations. Growth curves of bacteria around the expected MIC on the microdroplet array under exposure of A) amoxicillin, B) chloramphenicol and C) tetracycline in combination with streptomycin. D-F) FICI heatmaps for the respective combinations.

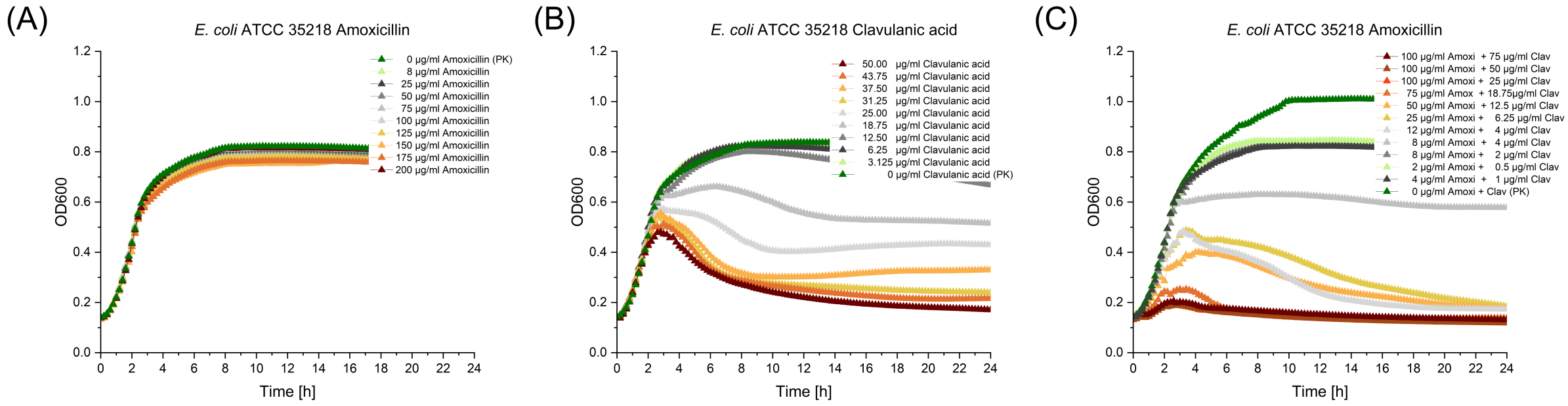

Figure S15. Growth of E. coli ATCC 35218 under varying concentrations of A) amoxicillin, B) clavulanic acid, C) and their combination, measured using a plate reader. The FICI derived from these experiments was 0.188, indicating a synergistic effect.

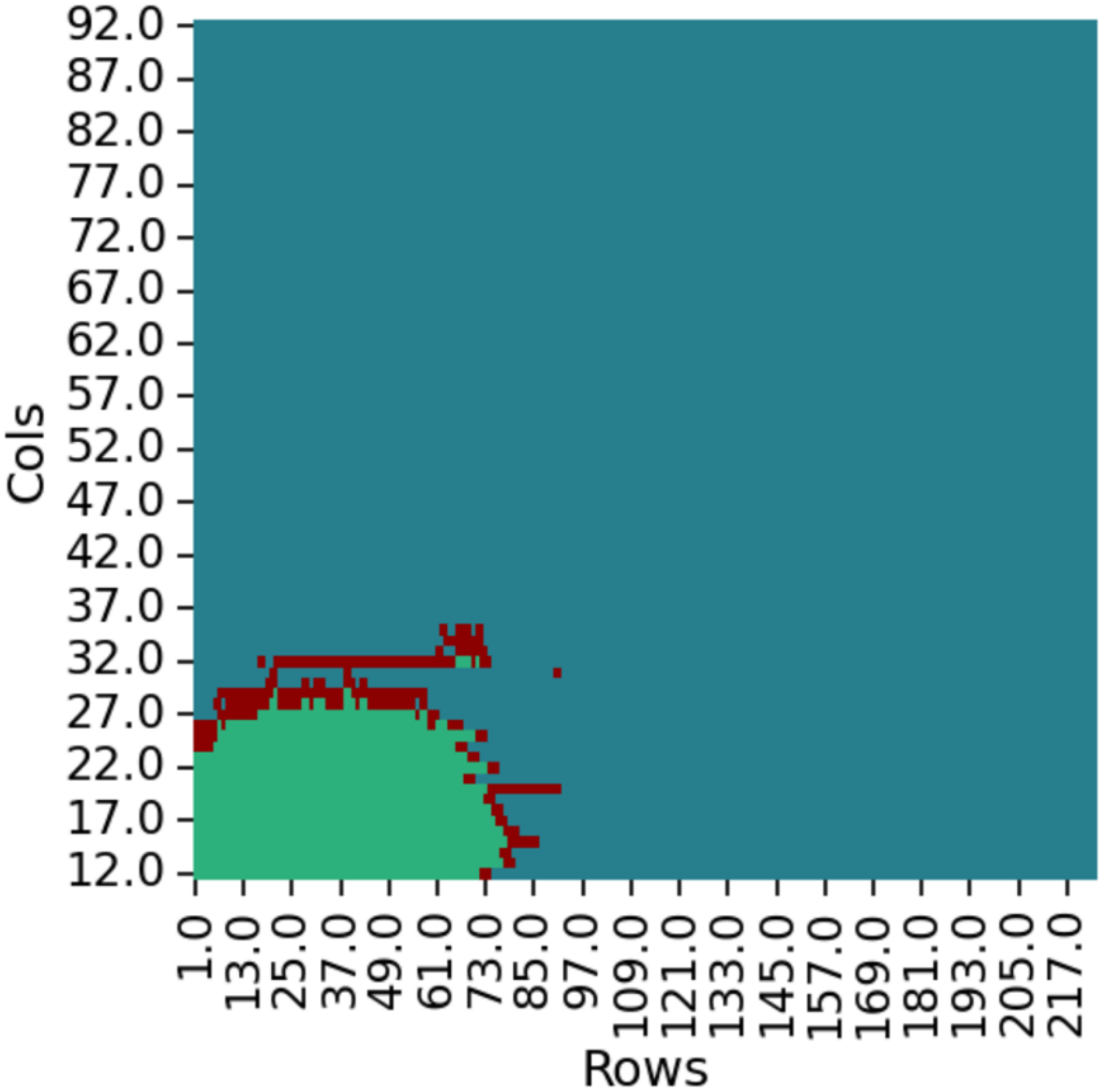

Figure S16. Classification of the bacterial states into dead, live and MIC of the amoxicillin/streptomycin combination obtained from the fluorescence data. Droplets were classified into growth and no-growth states based on an intensity threshold (LOD). The MIC transition region was defined by identifying the outer boundary of the growth region, corresponding to the highest antibiotic concentrations that still supported growth along each axis. These boundary points were assigned as a third state, resulting in a three-state map of inhibited, growing, and MIC-transition droplets.

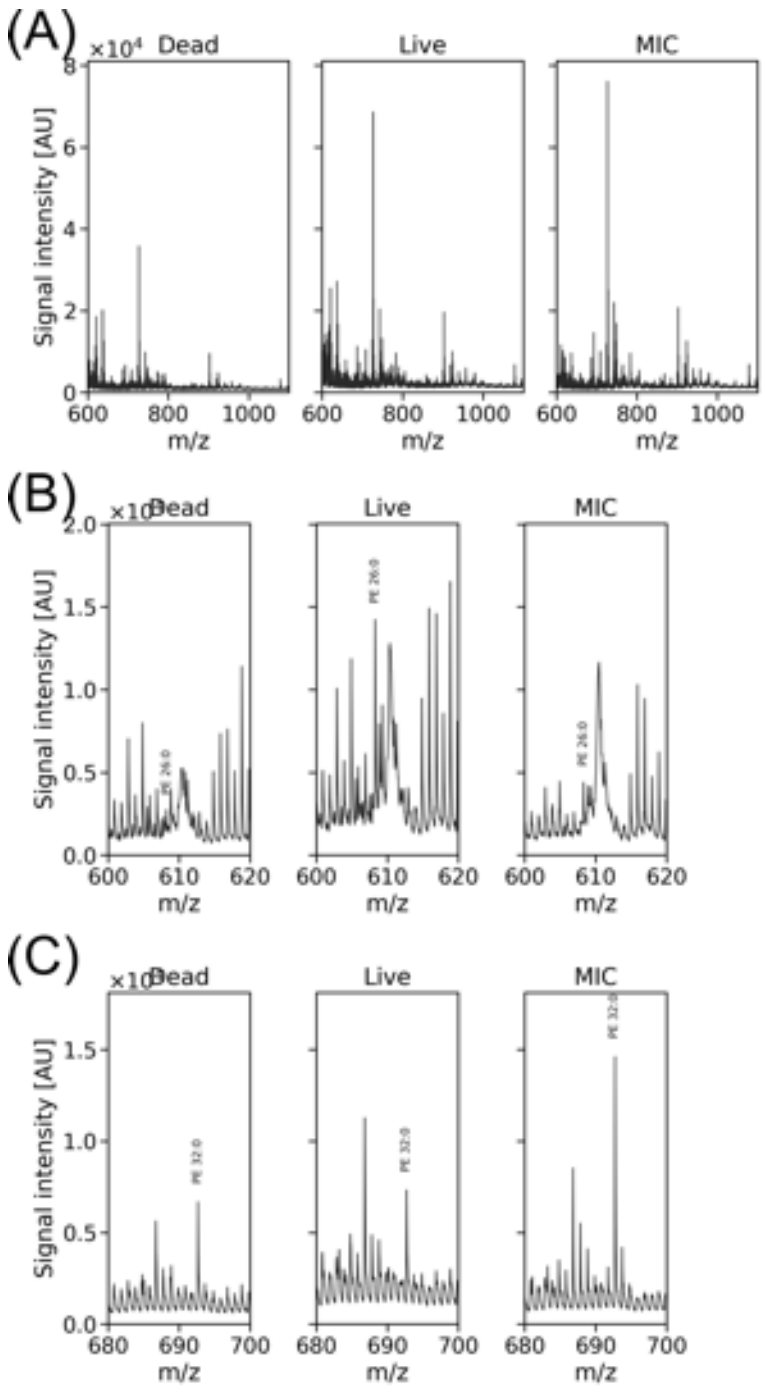

(D)

Input Mass	Matched Mass	Delta	Lipid	Formula	Ion
608.316	608.4286	0.1126	PE 26:0	C31H62NO8P	[M+H]+
692.647	692.5225	0.1245	PE 32:0	C37H74NO8P	[M+H]+
692.647	692.4262	0.2208	PE 31:4	C36H64NO8PNa	[M+Na]+
724.71	724.4912	0.2188	PE 35:5	C40H70NO8P	[M+H]+
724.71	724.4888	0.2212	PE 33:2	C38H72NO8PNa	[M+Na]+
725.69	725.4364	0.2536	PG 31:3	C37H67O10PNa	[M+Na]+
783.578	783.5171	0.0609	PG 37:5	C43H75O10P	[M+H]+
783.578	783.5147	0.0633	PG 35:2	C41H77O10PNa	[M+Na]+
903.65	903.6086	0.0414	PG 44:5	C50H89O10PNa	[M+Na]+
747.7285	747.5171	0.2114	PG 34:2	C40H75O10P	[M+H]+
749.744	749.5327	0.2113	PG 34:1	C40H77O10P	[M+H]+
749.744	749.4364	0.3076	PG 33:5	C39H67O10PNa	[M+Na]+
748.658	748.5851	0.0729	PE 36:0	C41H82NO8P	[M+H]+
748.658	748.4912	0.1668	PE 37:7	C42H70NO8P	[M+H]+
748.658	748.4888	0.1692	PE 35:4	C40H72NO8PNa	[M+Na]+
764.715	764.5225	0.1925	PE 38:6	C43H74NO8P	[M+H]+
764.715	764.5201	0.1949	PE 36:3	C41H76NO8PNa	[M+Na]+

Figure S17. Mass spectra and matched lipids. A) Full mass spectra in the mass range of the 10 most important mass peaks. B) Mass spectra of the peak at 692.647 m/z (PE 26:0) and C) of the peak at 608.316 m/z (PE 32:0) D) Table containing the matched lipids of the top 10 peaks.

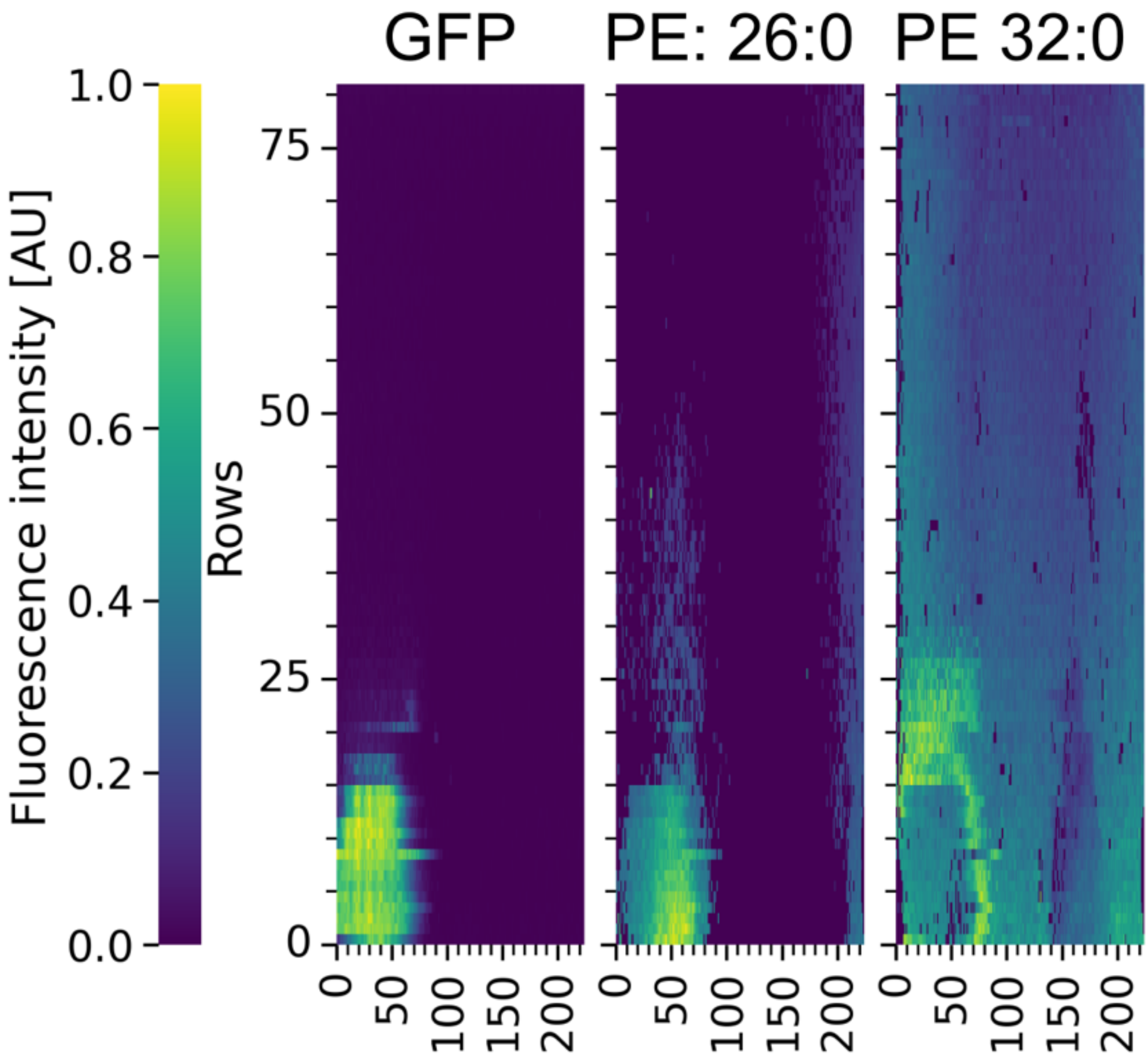

Figure S18. Side-by-side heatmaps of the fluorescence and MALDI-MS intensities.

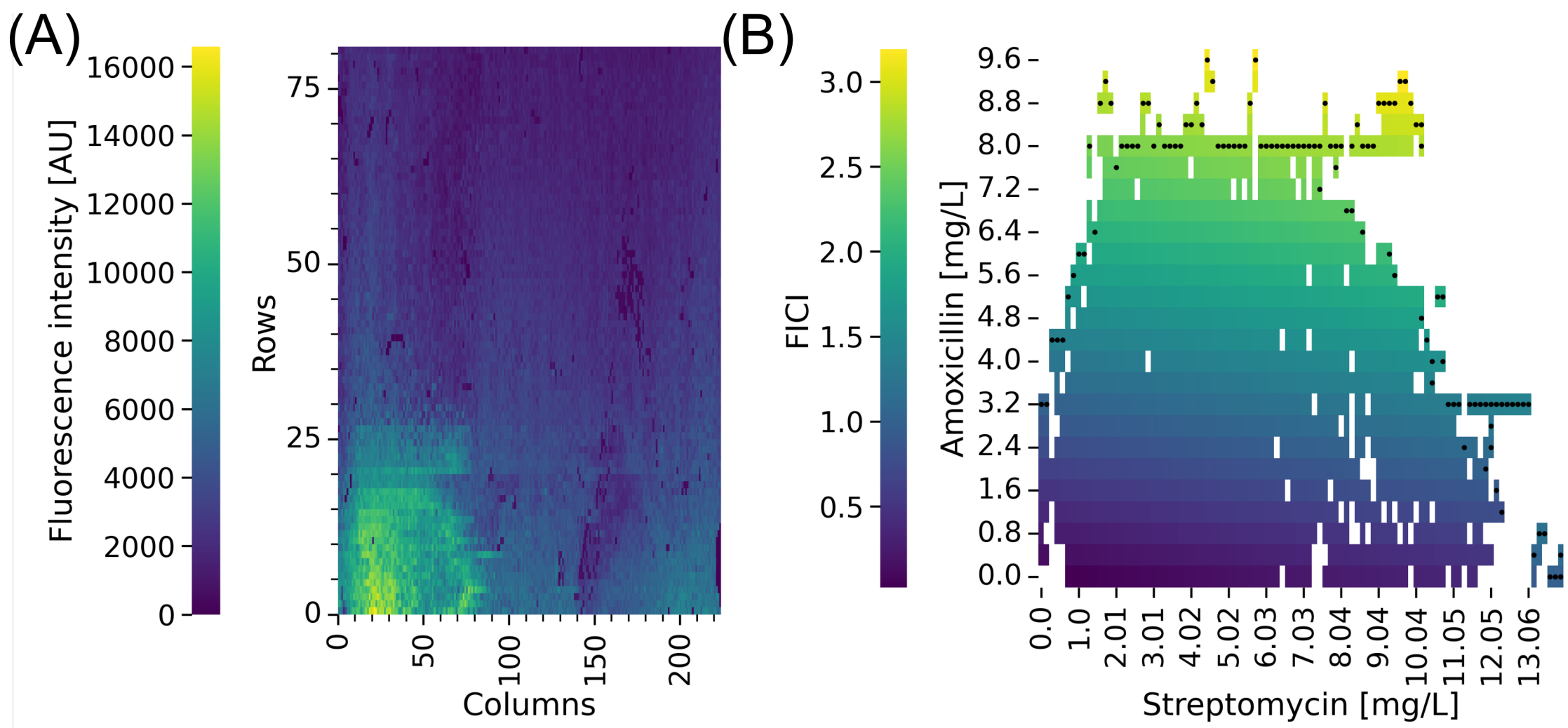

Figure S19. A) MALDI-MS heatmap showing the average intensity of the top 10 lipid features. B) Derived MIC values were used to calculate FICI indices, resulting in an average FICI of 2.21±0.73.

Table 1. List of reagents

Compound	Supplier	Catalog No.
Medium MHB II	Merck	90922
Kanamycin sulfate	Merck	PHR1487
Amoxicillin	Merck	A8523
Ampicillin	Carl Roth	K029.5
Carbenicillin	Carl Roth	6344.2
Chloramphenicol	Merck	C0378
Ciprofloxacin	Merck	PHR1044-1g
Meropenem	Merck	M2574-10MG
Polymixin B	Merck	P4932
Streptomycin	Carl Roth	0236.2
Tetracycline	Merck	7660-5G
Fluorescein	Merck	46955
Sulforhodamine B	Merck	230162
Dextran Cascade Blue (10K MW)	Life Technologies Europe B.V.	D1976
Dextran Alexa Fluor 647 (10K MW)	Life Technologies Europe B.V.	D22914
2,5 DHB	Merck	149357
